# Enhanced Antibacterial Properties of Lyotropic Liquid Crystalline Nanoparticles via Curvature Modulation

**DOI:** 10.1002/advs.76516

**Published:** 2026-07-09

**Authors:** Xiangfeng Lai, Shuhong Wang, Chenguang Ding, Xenia Kostoulias, Anton P. Le Brun, Hsien‐Yi Hsu, Jhih‐Hang Jiang, Yajun Wang, Richard A. Strugnell, Anton Y. Peleg, Hsin‐Hui Shen

**Affiliations:** ^1^ Department of Materials Science and Engineering Faculty of Engineering Monash University Melbourne Victoria Australia; ^2^ Department of Kidney Transplantation Nephropathy Hospital The First Affiliated Hospital of Xi'an Jiaotong University Xi'an Shaanxi P. R. China; ^3^ Institute of Organ Transplantation Xi'an Jiaotong University Xi'an P. R. China; ^4^ Infection and Immunity Program Monash Biomedicine Discovery Institute and Department of Microbiology Monash University Clayton Victoria Australia; ^5^ Australian Centre for Neutron Scattering Australian Nuclear Science and Technology Organisation Kirrawee DC New South Wales Australia; ^6^ School of Energy and Environment & Department of Materials Science and Engineering & Centre for Functional Photonics (CFP) City University of Hong Kong Kowloon Tong Hong Kong P. R. China; ^7^ Shenzhen Research Institute of City University of Hong Kong Shenzhen P. R. China; ^8^ College of Chemistry & Materials Engineering Wenzhou University Wenzhou Zhejiang P. R. China; ^9^ Department of Microbiology and Immunology at the Peter Doherty Institute for Infection and Immunity University of Melbourne Melbourne Victoria Australia; ^10^ Biomedicine Discovery Institute and Department of Biochemistry and Molecular Biology Monash University Melbourne Victoria Australia

**Keywords:** antimicrobials, Gaussian curvature, lyotropic liquid crystalline nanoparticles, nanostructures, structure‐activity relationship

## Abstract

Lyotropic liquid crystalline nanoparticles (LCNPs), including cubosomes, are increasingly investigated as antimicrobial nanomaterials because non‐lamellar lipid nanoparticles can fuse with biological membranes, exchange lipids, and improve antimicrobial delivery or antibiotic combination treatment. However, prior studies have mainly addressed fusion, uptake, encapsulation, or payload stabilization, rather than testing whether retained internal curvature can be isolated as a design variable for antibacterial potentiation in a matched LCNP series. Herein, we generated lamellar vesicles, primitive cubosomes (P‐cubosomes, *Im3m*), and diamond cubosomes (D‐cubosomes, *Pn3m*) from the same phytantriol/DPPS lipid system. When combined with free daptomycin, rather than being used as drug‐loaded carriers, these LCNPs exhibited curvature‐dependent potentiation hierarchy against methicillin‐resistant *Staphylococcus aureus* (MRSA), vesicles < P‐cubosomes < D‐cubosomes. Fluorescence imaging, electron microscopy, and neutron reflectometry showed progressively stronger membrane association, lipid extraction, and bilayer disruption with increasingly negative curvature. In a murine bacteremia model using a sub‐optimal daptomycin regimen, the same curvature‐dependent efficacy trend was retained in vivo, providing proof‐of‐concept support rather than therapeutic validation. This study provides direct experimental evidence, in a matched antibacterial LCNP system, that retained internal curvature modulates membrane remodeling and potentiates daptomycin against MRSA.

## Graphical abstract

Lipid nanostructured particles with increasing negative Gaussian curvature progress from vesicles to P‐cubosomes and D‐cubosomes. This curvature hierarchy enhances interaction with bacterial membranes, promotes membrane remodeling and leakage, and potentiates daptomycin activity against MRSA. D‐cubosomes produce the strongest membrane disruption, highlighting curvature as a tunable design parameter for membrane‐active antimicrobial nanomaterials.

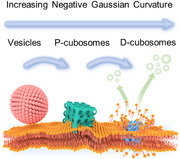

## 1 Introduction

Antimicrobial resistance (AMR) has emerged as a major global health crisis, with current estimates attributing 700 000 fatalities annually and projections reaching 10 million by 2050 without significant advances in antimicrobial therapies [1, 2, 3]. Nanomaterial‐based approaches are increasingly explored because their physicochemical properties can be engineered to modulate bacterial targeting, membrane interactions and therapeutic performance [4, 5]. Although parameters such as size, shape, and aspect ratio are well‐documented to affect antimicrobial activity [6, 7, 8], how internal nanoscale curvature contributes to antibacterial function remains less clearly defined.

**FIGURE 1:**
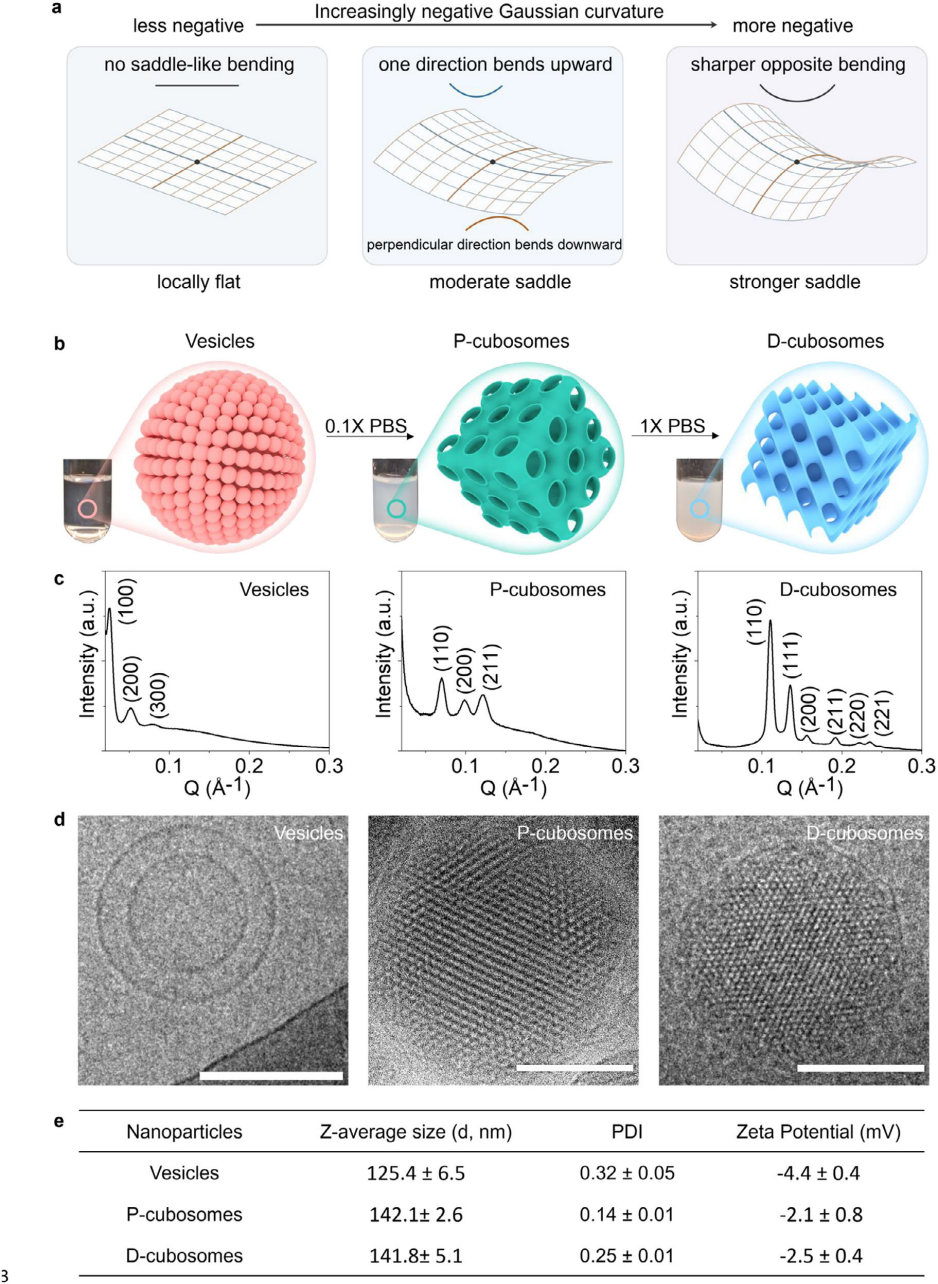
LCNP formulation, phase transition, and characterization. (a) Schematic of Gaussian curvature in LCNP surfaces. Negative Gaussian curvature means saddle‐like bending: upward in one direction and downward in the perpendicular direction. (b) Mixing 15 wt.% DPPS with phytantriol formed vesicles. Mixing vesicles with 0.1X PBS formed P‐cubosomes, further mixing with 1X PBS formed D‐cubosomes. (c) SAXS profiles for vesicles, P‐cubosomes and D‐cubosomes, along with assigned indices. a. u., arbitrary unit. (d) Representative Cryo‐TEM images of vesicles, P‐cubosomes, and D‐cubosomes. Scale bar: 100 nm. (e) The size and zeta potentials of LCNPs in cell culture media. Data points represent mean ± SD (*n* = 3).

At the level of membrane biophysics, membrane remodeling is governed by curvature elasticity, which defines the energetic landscape of processes such as fusion, pore formation, and lipid rearrangement [9, 10]. In particular, negative Gaussian curvature is associated with saddle‐shaped geometries that lower the energetic barrier for membrane remodeling and fusion intermediates (Figure 1a). These principles are directly relevant to nanoparticle‐membrane interactions, where internal structure can influence how lipid assemblies engage with biological membranes. Consistent with this, lipid nanoparticle structure has been shown to regulate membrane translocation processes, with non‐lamellar structures outperforming lamellar analogues [11]. Together, these findings establish curvature as a key determinant of membrane interaction.

Lyotropic liquid crystalline nanoparticles (LCNPs) therefore offer a suitable platform to test this principle, as changes in their internal phase structure correspond to distinct and well‐defined curvature regimes within the same lipid system. Experimental studies have shown that LCNPs can fuse with supported lipid bilayers and mammalian membranes, with fusion kinetics governed by internal structure [12]. At bacterial interfaces, nanoparticle interaction is strongly dependent on envelope architecture, with distinct uptake pathways observed across Gram‐positive and Gram‐negative systems [13], consistent with observations in fusogenic liposome systems [14]. These findings are consistent with a curvature‐dependent mechanism, in which differences in internal structure correspond to differences in membrane interaction behavior. Taken together, these findings indicate that curvature is expected to influence bacterial membrane interaction. However, most antibacterial LCNP studies have instead examined these systems in the context of drug delivery or cargo‐mediated activity, where improved efficacy is attributed to enhanced intracellular transport, protection of antimicrobial agents, or stabilization of peptide cargo [15, 16, 17, 18]. Additional studies further show that LCNP structure can be dynamically reconfigured through interactions with membrane‐active molecules [19, 20, 21, 22]. While these studies demonstrate that LCNPs are both membrane‐active and antibacterial, their effects arise from multiple co‐varying factors, including payload, composition, and environmental conditions, and therefore do not isolate curvature as an independent structural parameter governing antibacterial potentiation.

Here, we address this limitation by developing a curvature‐programmed LCNP platform comprising lamellar vesicles, primitive cubosomes (*Im3m*), and diamond cubosomes (*Pn3m*) derived from the same phytantriol/DPPS system. After structural verification, all formulations were transferred into the same final assay medium, where their internal phases remained stable over the experimental timescale. Using this matched system, we evaluate whether blank LCNPs, when co‐administered with free daptomycin rather than used as drug carriers, can modulate antibacterial activity against methicillin‐resistant *Staphylococcus aureus* (MRSA). By combining microbiological assays, imaging, neutron reflectometry, and an in vivo bacteremia model, we demonstrate that progressively more negative curvature is associated with progressively stronger membrane remodeling and antibiotic potentiation, providing direct experimental evidence that retained internal curvature is a controllable design parameter for antibacterial nanomaterials.

## 2 Results

### 2.1 Characterization and Phase Transition of LCNPs

15 wt.% DPPS was mixed with the phytantriol to induce lamellar vesicles (Figure S1). Remarkably, the system underwent a spontaneous structural transition from lamellar vesicles to P‐cubosomes and subsequently to D‐cubosomes upon the sequential addition of 0.1X and 1X PBS, respectively, as characterized by SAXS and cryo‐TEM analyses (Figure 1b–d). After confirmation of phase structure, all LCNP formulations were diluted into the same final biological assay medium before physicochemical and biological measurements. SAXS collected after 24 h in the assay medium (with or without co‐administration with daptomycin) retained the characteristic Bragg reflections for all three LCNPs (Figures S2 and S3), confirming structural persistence over the assay timescale.

Despite their distinct internal geometries, all three LCNP formulations displayed comparable Z‐average hydrodynamic sizes and zeta potentials when dispersed in the same biological assay medium. Dynamic light scattering in the assay medium yielded particle diameters of 125.4 ±  6.5 nm for vesicles, 142.1 ± 2.6 nm for P‐cubosomes, and 141.8 ± 5.1 nm for D‐cubosomes, with a near‐neutral zeta potential of around −3 mV (Figure 1e, Figure S4). Comparable values were obtained in the CaCl_2_/NaCl/ HEPES buffer used for neutron reflectometry (Figure S5 and Table S1), indicating consistent colloidal properties across measurement conditions. Importantly, co‐administration with daptomycin did not substantially alter the relative size or apparent zeta‐potential profiles of the three LCNP formulations under the same final assay conditions (Table S2). Thus, the LCNPs remained comparable in average hydrodynamic size and apparent electrokinetic properties before and after daptomycin co‐administration, allowing subsequent comparisons to focus on differences in retained internal nanostructure.

Although each nanoparticle presents the same DPPS‑phytantriol outer leaflet, only the cubic phases expose saddle‑shaped openings where the bicontinuous water channels meet the surface, whereas the vesicle surface is topologically flat. Applying the Gauss‐Bonnet theorem to the SAXS‐derived lattice parameters gives a Gaussian curvature of 0 nm^−2^ for vesicles, −0.07 ± 0.01 nm^−2^ for P‐cubosomes, and −0.10 ± 0.01 nm^−2^ for D‐cubosomes (Table S3) [23, 24, 25]. Increasing PBS strength screens head‐group repulsion, reduces interfacial area, and raises the packing parameter, driving the system toward phases with progressively more negative curvature [23, 26, 27, 28, 29]. This compaction accounts for both the observed lattice contraction and the higher curvature stress in the cubic particles (Figure 1d, Table S3), establishing a well‐defined curvature hierarchy across the LCNP series for subsequent analysis.

### 2.2 LCNPs Exhibit Transitional Membrane‐Interaction and Antimicrobial Activity

**FIGURE 2:**
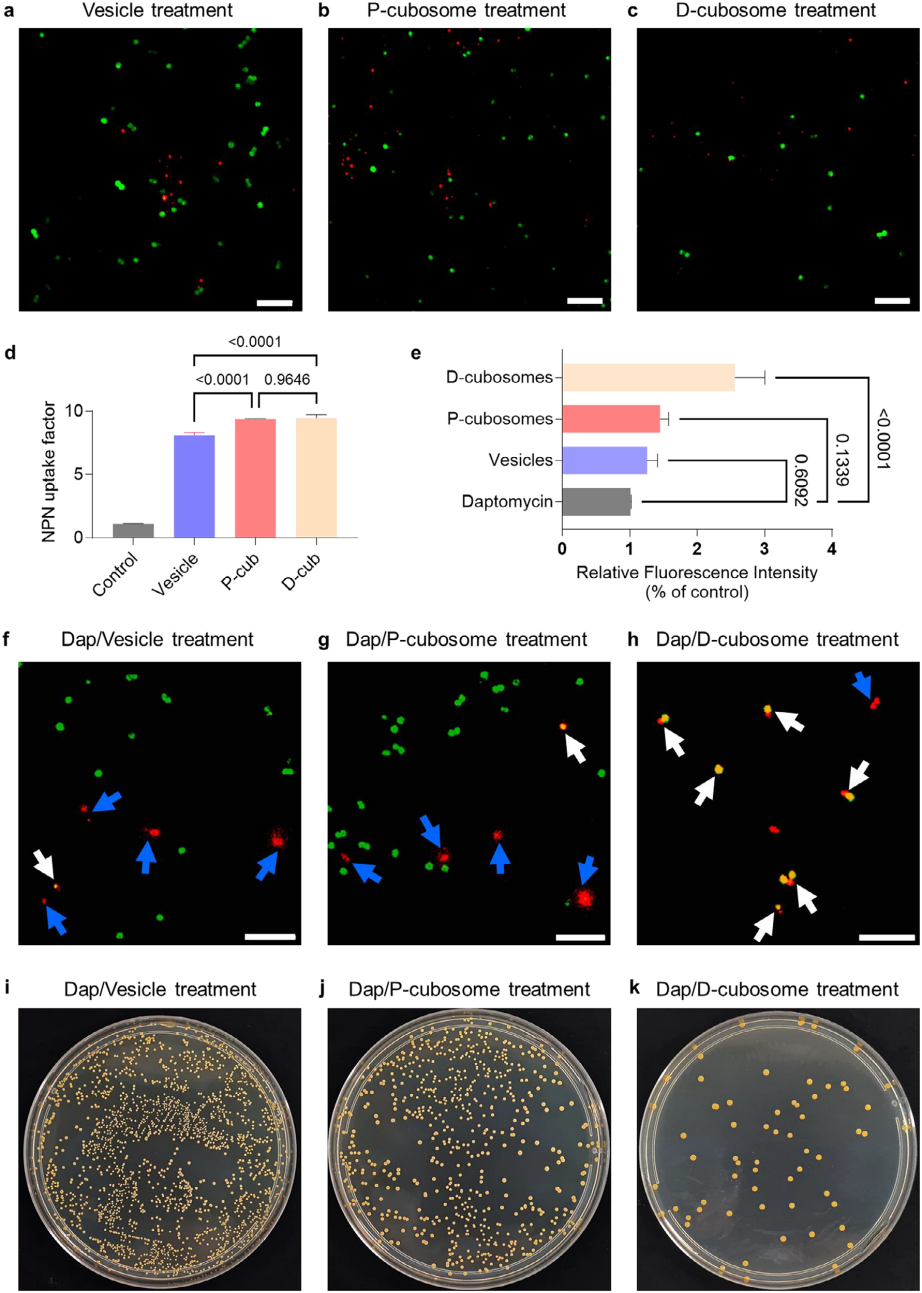
LCNP‐membrane interaction. Fluorescence microscopy images of MRSA A8819 under monotherapies with (a) Vesicles, (b) P‐cubosomes, and (c) D‐cubosomes at 64 ug/mL. (d) MRSA A8819 membrane permeabilization as measured with 1‐N‐phenylnaphthylamine (NPN) fluorescence emission (Ex/Em: 340/405 nm). Data points represent mean ± SD (*n* = 3). (e) Oxidative stress in MRSA A8819 measured by relative fluorescence intensity at 24 h, using 2',7'‐dichlorofluorescin diacetate (DCFDA) staining under different LCNP treatments at 64 µg/mL. Error bars represent the mean ± SD (*n* = 3). Combination treatments of (f) Daptomycin (Dap)/vesicle, (g) Dap/P‐cubosomes, and (h) Dap/D‐cubosomes. Scale bar: 5 µm. The MRSA membrane was stained with CellBrite Fix 488 (green) and LCNPs with Rhodamine B (red) in all images. (i–k) Representative images of MRSA A8819 grown on agar plates after combination treatments of daptomycin/LCNPs. Statistical significance was determined using one‐way ANOVA.

The interactions between LCNPs and the membranes of six MRSA strains were investigated using confocal laser scanning microscopy, with consistent results observed across all strains (Figure 2a–c, Figure S6). In monotherapy against MRSA A8819, all three fluorescently labeled LCNPs (in red) were observed in close proximity to the bacterial membrane (in green) (Figure 2a–c). We then performed the 1‐N‐phenylnaphthylamine (NPN) uptake assay to quantify LCNP interaction with the bacterial membrane [30, 31, 32]. The results revealed a concentration‐dependent increase in NPN fluorescence for all three LCNPs (Figure 2d, Figure S7a). At 64 µg/mL, D‐cubosomes exhibited the highest NPN uptake factor (9.5), followed closely by P‐cubosomes (9.4), while vesicles showed lower uptake (8.1). This trend was consistent across all tested concentrations (16–64 µg/mL), with D‐cubosomes maintaining the greatest interaction with the membrane hydrophobic region. The effects of LCNP monotherapy on bacterial membrane permeability were further explored using 2',7'‐dichlorodihydrofluorescein diacetate (Figure 2e, Figure S7b). Compared with daptomycin‐treated controls, LCNPs induced oxidative stress in a concentration‐dependent manner. Among the LCNP monotherapy, D‐cubosomes triggered the most pronounced oxidative stress, increasing fluorescence intensity by over 2.6%, compared with 1.4% and 1.2% for P‐cubosomes and vesicles, respectively.

However, our previous findings suggest that membrane‐targeting antibiotics, such as polymyxin, can destabilize bacterial membranes and facilitate nanoparticle interaction [21]. Building on this principle, we explored daptomycin, a membrane‐targeting antibiotic active against Gram‐positive bacteria, to enhance LCNP activity against MRSA. By disrupting membrane integrity through interaction with negatively charged bacterial surfaces, daptomycin increases membrane permeability [33, 34], which may facilitate LCNP engagement. To assess the combined action between free daptomycin and LCNPs (daptomycin/LCNP combination treatment), subinhibitory concentrations of daptomycin were used to minimize resistance risk while evaluating LCNP interaction with MRSA membranes (Figure 2f–h). The addition of free daptomycin in vesicle and P‐cubosome combinations led to limited membrane association (Figure 2f,g, white arrow), with many nanoparticles aggregating without extensive membrane engagement (Figure 2f,g, blue arrow). This limited interaction is consistent with previous findings that daptomycin combinations do not always enhance therapeutic efficacy [35]. In contrast, the daptomycin/D‐cubosome combination (Figure 2h) showed clear colocalization (yellow regions, white arrows) and direct membrane interaction, indicating stronger membrane engagement compared to other formulations. To validate these findings, minimum inhibitory concentration (MIC) assays were conducted in MHB media or HEPES (Figure S8), following Infectious Diseases Society of America recommendations [36]. The results showed that ionic strength alone did not alter daptomycin activity, while combining daptomycin with LCNPs reduced MIC values in MRSA A8819 by eight‐fold with vesicles, 32‐fold with P‐cubosomes, and 64‐fold with D‐cubosomes compared with LCNP monotherapy (MIC > 512 µg/mL). These control experiments indicate that ionic strength alone does not explain the observed enhancement in daptomycin activity. A similar trend was consistent across other MRSA strains, consistent with a curvature‐dependent potentiation hierarchy (vesicles < P‐cubosomes < D‐cubosomes) (Figure 2i–k). Although these fold reductions in MIC are substantial, FICI values (Tables S4–S6) remain within the conventional indifferent, non‐synergistic range across the six tested MRSA strains (vesicles: 0.531–0.750; P‐cubosomes: 0.508–0.625; D‐cubosomes: 0.504–0.563), with D‐cubosomes consistently at the lower end, indicating curvature‐dependent potentiation rather than formal pharmacodynamic synergy.

The wild‐type MRSA membrane, predominantly composed of lysyl‐phosphatidylglycerol, phosphatidylglycerol, and cardiolipin at a molar ratio of 19:69:12, has a natural bias toward positive curvature due to the high proportion of lysyl‐phosphatidylglycerol [34, 37, 38]. Upon interaction with the bacterial membrane, D‐cubosomes, which possess a higher negative Gaussian curvature, are associated with stronger membrane perturbation compared to vesicles and P‐cubosomes. This observation is consistent with curvature mismatch between the nanoparticle and the bacterial membrane, which has been proposed to generate localized stress and promote membrane destabilization [39, 40]. Such curvature‐driven effects are known to lower the energetic barrier for membrane remodeling processes, including fusion and lipid rearrangement [10], and are consistent with an increased susceptibility of the membrane to structural rearrangement [41]. As a result, D‐cubosomes exhibit enhanced membrane interaction relative to other LCNP formulations, correlating with their increased antibacterial potentiation in combination with daptomycin [42].

### 2.3 LCNPs Induce Transitional Membrane Disruption

**FIGURE 3:**
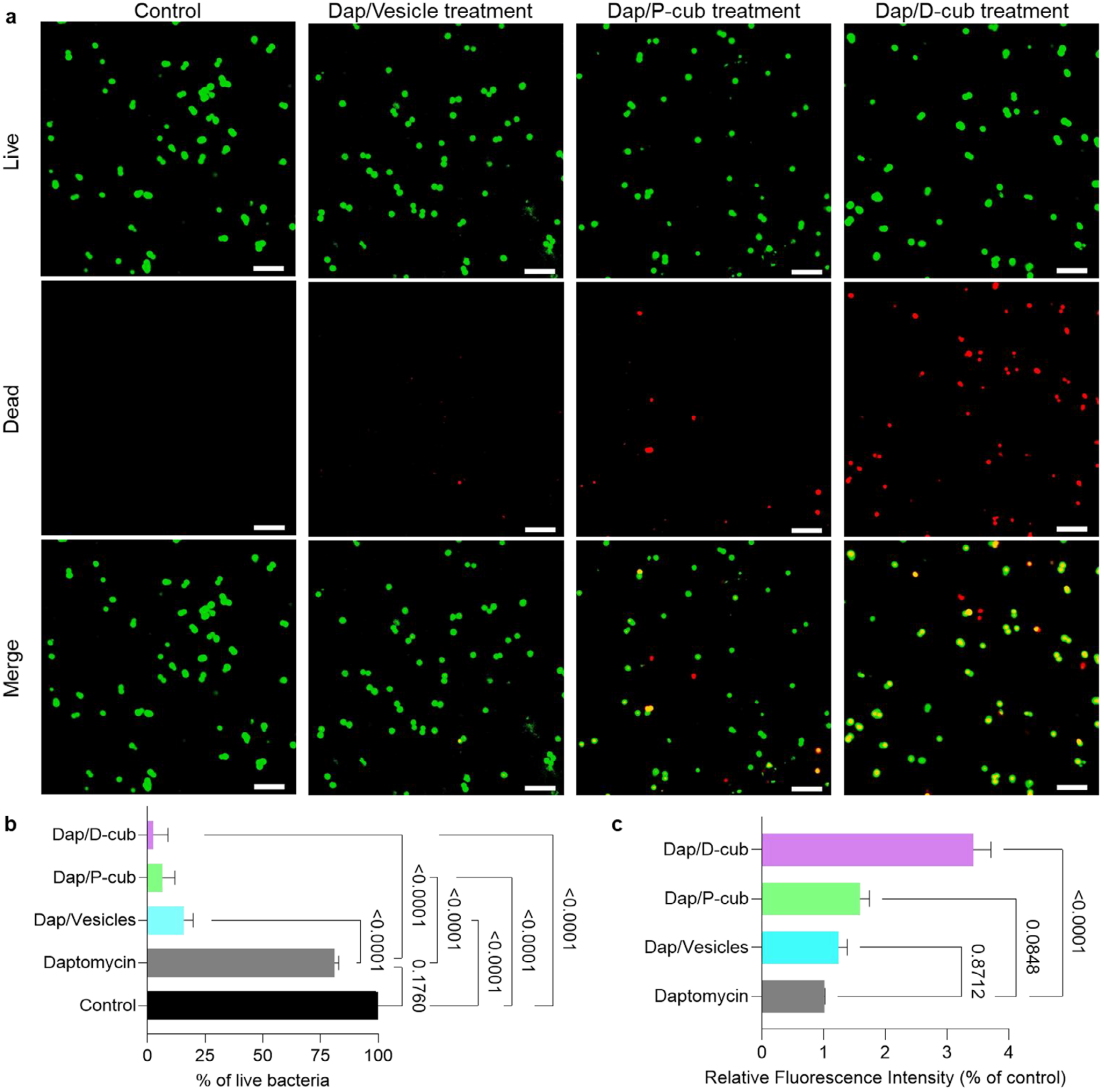
Fluorescence study on the interaction of LCNPs with MRSA A8819. (a) Representative live/dead staining images of MRSA A8819 (control) and treatments with daptomycin (Dap)/vesicles, Dap/P‐cubosomes, and Dap/D‐cubosomes. Scale bars, 5 µm. (b) Quantification of MRSA A8819 viability (*n* = 3) under various treatments over 4 h at 37°C using a fluorescence microplate reader. (c) Oxidative stress in MRSA A8819 was measured by relative fluorescence intensity at 24 h, using 2',7'‐dichlorofluorescin diacetate (DCFDA) staining under different treatment conditions. Daptomycin (Dap, 0.25 µg/mL), LCNPs: 64 µg/mL. Error bars represent the mean ± SD (*n* = 3). Statistical significance was determined using one‐way ANOVA.

The antimicrobial assessment was further complemented by a membrane integrity analysis utilizing a live/dead staining assay (Figure 3a), which distinguishes viable from nonviable cells through the differential uptakes of SYTO9 and propidium iodide (PI), where SYTO9 produces green fluorescence within live and dead cells as it is membrane permeable, and PI emits red fluorescence upon intercalating with DNA from cells with compromised membranes, indicative of cell death [43]. Monotherapy of daptomycin was predominantly characterized by green fluorescence, signifying a majority of live cells (Figure S9). Notably, control experiments with LCNP‐only treatments using SYTO 9/propidium iodide staining showed negligible fluorescence changes in the absence of membrane disruption, indicating minimal interference of LCNPs with fluorescence‐based viability dyes. Upon combined treatment with daptomycin, vesicles displayed insufficient bactericidal activity, as evidenced by the lack of significant PI uptake, indicating maintained membrane integrity (Figure 3a). Conversely, P‐cubosomes caused mild membrane disruption, observable through moderate PI permeation (red fluorescence). In stark contrast, treatment with D‐cubosomes led to an extensive PI uptake, signifying severe membrane compromise and elevated cell mortality. These findings indicate differential membrane destabilization capabilities of LCNPs across different phases, with D‐cubosomes exhibiting a pronounced ability to potentiate the bactericidal effects of daptomycin, consistent with the membrane interaction and antimicrobial efficacy previously noted in Figure 2h,k.

To quantify these observations, bacterial viability was analyzed under each treatment condition using SYTO 9 and PI staining, which differentiate live and dead bacteria based on membrane integrity (Figure 3b, Figure S10). Daptomycin monotherapy resulted in minimal membrane disruption, leaving approximately 81% of bacteria viable, comparable to the untreated control. In combination treatments, the daptomycin/D‐cubosome combination produced the most pronounced bactericidal activity, reducing bacterial viability to below 2%. Combinations involving daptomycin with P‐cubosomes and vesicles also showed increased bactericidal activity, reducing viability to approximately 7% and 15%, respectively, though they were less effective than the daptomycin/D‐cubosome combination. These results quantitatively support enhanced membrane disruption and antibacterial efficacy of daptomycin/D‐cubosome, consistent with a curvature‐dependent trend in antimicrobial activity.

Further, daptomycin/D‐cubosomes triggered the most pronounced oxidative stress by approximately 3.6% (Figure 3c). In contrast, daptomycin/P‐cubosome and daptomycin/vesicle combinations exhibited less pronounced effects, with fluorescence intensities ranging between 1.2 and 1.6%. These findings indicate that the daptomycin/D‐cubosome induces greater oxidative stress and membrane disruption compared to other treatments. This combined action likely arises from the interplay of D‐cubosomes’ sharp negative Gaussian curvature and daptomycin's ability to increase membrane permeability at sub‐MIC levels [44]. Together they impose significant localized stress on bacterial membranes, weakening their structural integrity and enhancing susceptibility to disruption. This curvature‐driven potentiation aligns with the previously discussed curvature mismatch mechanism, where the high Gaussian curvature of D‐cubosomes imposes significant energetic stress on Gram‐positive membranes, weakening their structural integrity and making them more susceptible to disruption.

**FIGURE 4:**
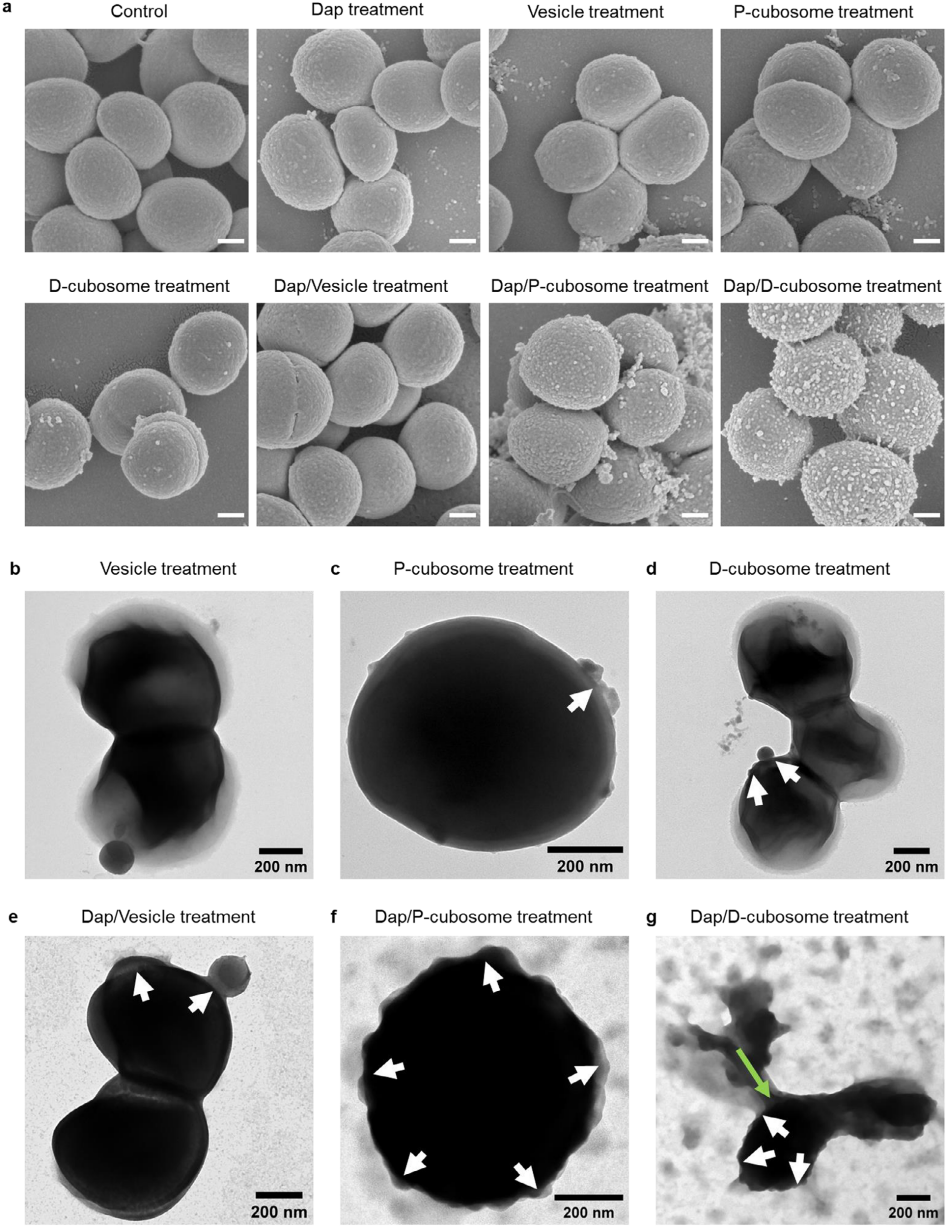
Morphological studies of MRSA A8819 by electron microscopy. (a) SEM images of MRSA A8819 (control) and with different treatments. Scale bar: 500 nm. TEM images of MRSA A8819 treated with (b) Vesicle, (c) P‐cubosomes, (d) D‐cubosomes, (e) Dap/vesicles, (f) Dap/P‐cubosomes and (g) Dap/D‐cubosomes. Regions of interest are marked with white and green arrows, with the corresponding areas enlarged and displayed in Figure S11g–l for detailed visualization.

Subsequent SEM images (Figure 4a) provided visual confirmation of the bacterial membrane impact induced by LCNPs, both alone and in combination with daptomycin. The morphological changes observed post‐treatment reveal membrane interaction and disruption. Prior to any treatment (control), MRSA A8819 cells presented a uniform and smooth surface morphology. Post‐treatment with daptomycin at 0.5 × MIC, the cells retained their structural integrity, suggesting that daptomycin's mechanism of action at this concentration does not prominently involve disruption of cell morphology or surface integrity. Consistently, the application of each LCNP did not result in observable morphological changes, indicating that LCNPs alone are insufficient to induce detectable membrane perturbation under these conditions. In line with the fluorescence microscopic result, daptomycin/vesicle did not induce morphological changes. In contrast, daptomycin/P‐cubosomes had limited impact on the bacterial surface and caused moderate membrane blebbing. Remarkably, significant membrane disruption in the presence of daptomycin/D‐cubosomes was observed. Specifically, this treatment resulted in the formation of multiple “bread crumb”‐like protrusions on the surfaces of treated cells, while the untreated cells and other treatments lacked these features. These morphological features are consistent with enhanced membrane perturbation in the presence of D‐cubosomes. Previous theoretical and simulation works suggest that increasing negative Gaussian curvature can lower the energy barrier for pore formation [45], and that cubic lipid nanostructures may facilitate lipid exchange during membrane interaction [21]. Accordingly, the observed membrane damage in the daptomycin/D‐cubosome treatment is consistent with curvature‐enhanced membrane disruption. Semi‐quantitative analysis of SEM images confirmed this trend, with the daptomycin/D‐cubosome treatment showing the highest proportion of damaged cells (Figure S11a). The absence of comparable damage in vesicle/drug or daptomycin‑only controls highlight the need for both membrane perturbation and nanoparticle interaction, accounting for the enhanced antibacterial activity of the D‑cubosome combination. This observation is consistent with the fluorescence results shown in Figure 2h and Figure 3a, suggesting that the interaction between D‐cubosomes and bacterial membranes contributes to the observed morphological changes.

Next, we used conventional TEM to visualize LCNP morphology and MRSA envelope changes after treatment (Figure 4b–g, Figure S11b–l). Because these images were acquired by conventional TEM, they were used to assess particle morphology and bacteria–particle contact, whereas hydrated internal LCNP structures were assigned from SAXS and cryo‐TEM (Figure 1b–d). Initially, TEM images revealed distinct morphological features among the three LCNPs formulations (Figure S11b–d). Vesicles showed a simple spherical structure with limited internal contrast, whereas P‐cubosomes and D‐cubosomes showed progressively more structured morphologies, consistent with their SAXS‐assigned internal phases. Prior to treatment, MRSA cells exhibited intact envelopes (Figure S11e). Daptomycin alone at 0.5 × MIC did not visibly affect envelope integrity (Figure S11f). Vesicles remained adjacent to the bacterial surface with limited interaction (Figure 4b, Figure S11g), while P‐cubosomes showed closer surface association (white arrow, Figure 4c, Figure S11h). D‐cubosomes showed the strongest bacterial surface contact and local membrane deformation (white arrow, Figure 4d, Figure S11i), suggesting greater membrane stress without immediate membrane rupture.

In combination with daptomycin, LCNP–bacteria interactions were enhanced. Vesicles showed increased surface adhesion (white arrows, Figure 4e, Figure S11j), while P‐cubosomes were more frequently observed around bacterial cells and in close contact with the envelope (white arrows, Figure 4f, Figure S11k). The daptomycin/D‐cubosome combination produced the most pronounced effect, with strong particle–cell association (white arrows, Figure 4g, Figure S11l) and visible cell lysis (green arrow, Figure 4g). Post‐mixing DLS and zeta‐potential measurements further showed that co‐administration with daptomycin did not introduce major formulation‐specific differences in hydrodynamic size, PDI, or apparent zeta potential (Table S2). Thus, the enhanced membrane disruption observed for D‐cubosomes is unlikely to arise from post‐mixing size or electrokinetic differences, and instead remains consistent with the stronger membrane interaction associated with retained internal nanostructure.

These TEM observations are consistent with previous model‐membrane studies showing that cubosomes can interact with lipid bilayers and promote lipid exchange or bilayer destabilization [12, 21, 46]. In the present system, conventional TEM cannot determine daptomycin embedding or stable daptomycin–LCNP complex formation. However, together with SAXS, post‐mixing DLS/zeta measurements and fluorescence/SEM assays, the TEM data support a co‐administration mechanism in which daptomycin may increase membrane susceptibility while LCNP curvature governs the extent of membrane interaction and disruption.

### 2.4 Biocompatible LCNPs are Effective In Vivo

**FIGURE 5:**
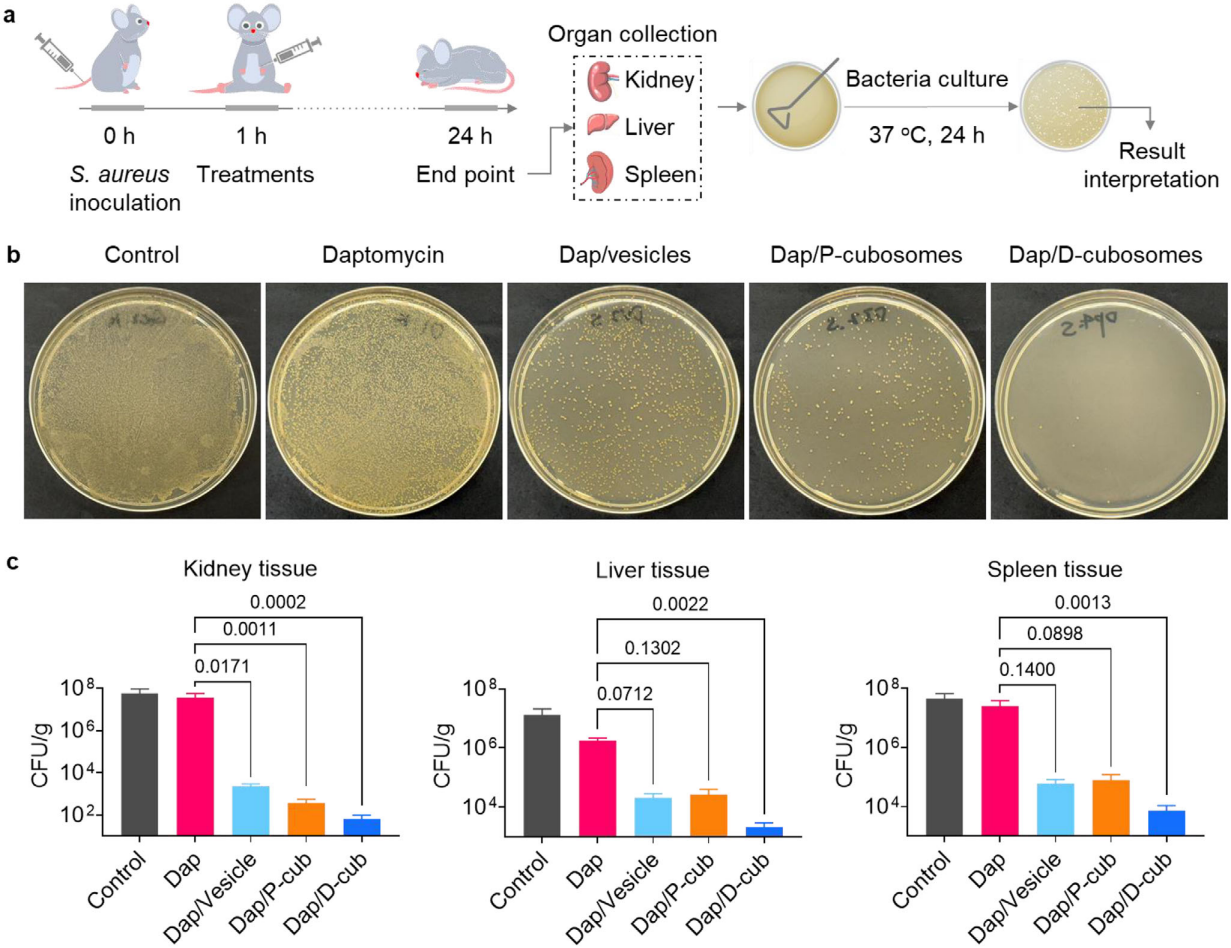
Proof‐of‐concept in vivo potentiation of daptomycin by LCNPs in a murine *S. aureus* bacteremia model. a Male and female BALB/c mice (*n*  =  5 per group) were inoculated with *S. aureus* via tail‑vein injection, then treated intraperitoneally 1 h later with: physiological saline (control), free daptomycin (10 mg kg^−^^1^), blank LCNPs (45.6 mg kg^−^^1^ lipid), or daptomycin/LCNPs (at equivalent doses). (b) Representative homogenates of organs (kidney, liver, and spleen) of mice after tail injection with saline (control), daptomycin (dap), dap/vesicles, dap/P‐cubosomes, and dap/D‐cubosomes for 24 h cultured on the solid MHB agar. **c** Quantified CFU of *S. aureus* in excised organ tissues from treatment endpoints was performed (*n* = 5). Data are presented as means ± sem. Statistical significance was determined using one‐way ANOVA.

To investigate biocompatibility, human HEK 293T cells were incubated for 24 h with vesicles, P‑cubosomes, or D‑cubosomes either alone or combined with sub‑MIC daptomycin (0.25 µg/mL). Metabolic activity, quantified via MTT assay, remained ≥  80 % for all treatments (Figure S12). Thus, the LCNP formulations and their combinations with daptomycin are non‑toxic toward mammalian cells under the tested conditions. To determine whether the curvature‑dependent effects observed in cell and membrane assays manifest in vivo, we challenged BALB/c mice intravenously with *S. aureus* and assessed bacterial loads in kidney, liver and spleen 24 h later (Figure 5a,b, Figure S13). Because LCNP phase behavior can be sensitive to ionic environment, the in vivo efficacy data were interpreted together with SAXS and post‐mixing DLS/zeta controls showing that the formulations retained their assigned internal structures and comparable colloidal properties after incubation in the final biological medium, with or without daptomycin co‐administration (Figure S2 and S3 and Table S2). Saline‑treated controls showed heavy infection, with median counts ≥ 10^7^ colony‐forming units (CFU)/g in all three organs and confluent growth on recovery plates (Figure 5b, Figure S13a). Administration of free daptomycin (10 mg/kg), selected to represent a sub‐optimal therapeutic condition enabling evaluation of LCNP‐mediated potentiation, did not significantly reduce bacterial burdens, with organ counts statistically indistinguishable from the saline group (Figure 5b, Figure S13b). Blank LCNPs (45.6 mg/kg) produced a modest yet consistent ∼2 log10 drop in CFU (*P* < 0.05 versus saline, Figure S13c–e,i).

When daptomycin was co‑administered with LCNPs (Figure 5c), a curvature‐dependent trend in bacterial clearance was observed, consistent with in vitro results. Combination with vesicles reduced organ burdens by an additional ∼2 log10, yielding residual loads of ∼10^5^ CFU/g (Figure S13f). Pairing daptomycin with P‑cubosomes further lowered counts to 10^3^–10^4 ^CFU/g (Figure S13g). The greatest reduction was observed with D‑cubosomes, where bacterial counts approached the detection limit of 10^2^ CFU/g, corresponding to a 5–6 log10 improvement over free daptomycin (Figure S13h). These in vivo results mirror the hierarchy observed in membrane interaction and antimicrobial assays, with D‐cubosomes showing the strongest potentiation, followed by P‐cubosomes and vesicles. The agreement between these mechanistic data and the animal outcomes indicates that the curvature‐dependent efficacy trend observed under structurally verified assay conditions is preserved in this proof‐of‐concept bacteremia model. The in vivo experiment focuses on establishing a proof‐of‐concept efficacy trend, not as therapeutic validation or evidence of preclinical readiness, and therefore does not include pharmacokinetic, long‐term toxicity, or survival analyses.

### 2.5 Neutron Reflectometry Reveals the Interaction Mechanism

While membrane disruption has been visualized, neutron reflectometry provides Ångström‐level structural details on how daptomycin and LCNPs remodel membrane lipid architecture [21, 34, 43, 46, 47, 48, 49, 50]. However, the native *S. aureus* envelope also contains a peptidoglycan‐rich cell wall above the cytoplasmic membrane, we first examined whether LCNP association was dominated by this outer layer using 7‐hydroxycoumarin carbonyl amino‐D‐alanine (HADA)‐labeled peptidoglycan and rhodamine B‐labeled LCNPs (Figure S14) [51]. The limited overlap between these signals indicated that LCNP association was not primarily localized to the peptidoglycan layer, supporting the use of a simplified lipid‐bilayer model to isolate the downstream membrane‐remodeling step. We therefore recreated the *S. aureus* cytoplasmic membrane with 1‐palmitoyl‐2‐oleoyl‐sn‐glycero‐3‐phospho‐(1'‐rac‐glycerol) (sodium salt) (PG), 1',3'‐bis[1,2‐dioleoyl‐sn‐glycero‐3‐phospho]‐glycerol (sodium salt) (CL) and 1,2‐dioleoyl‐sn‐glycero‐3‐[phospho‐rac‐(3‐lysyl(1‐glycerol))] (chloride salt) (L‐PG) at a 69/12/19 mol.% ratio, an established system previously validated for studying daptomycin–membrane interactions [34]. Such a model is used for studying membrane‐targeting antibiotics like daptomycin, and here we extend this approach to a co‐administration system with LCNPs. This enables direct assessment of how LCNP internal nanostructure influences daptomycin–membrane interactions. The simplified system allows quantitative resolution of membrane‐specific structural changes, including lipid loss, hydration, and bilayer disruption, which would be difficult to extract in more complex envelope models. Accordingly, daptomycin concentration was held constant, while only LCNP internal structure (vesicles, *Im3m*, *Pn3m*) was varied to directly assess curvature‐dependent membrane remodeling.

**FIGURE 6:**
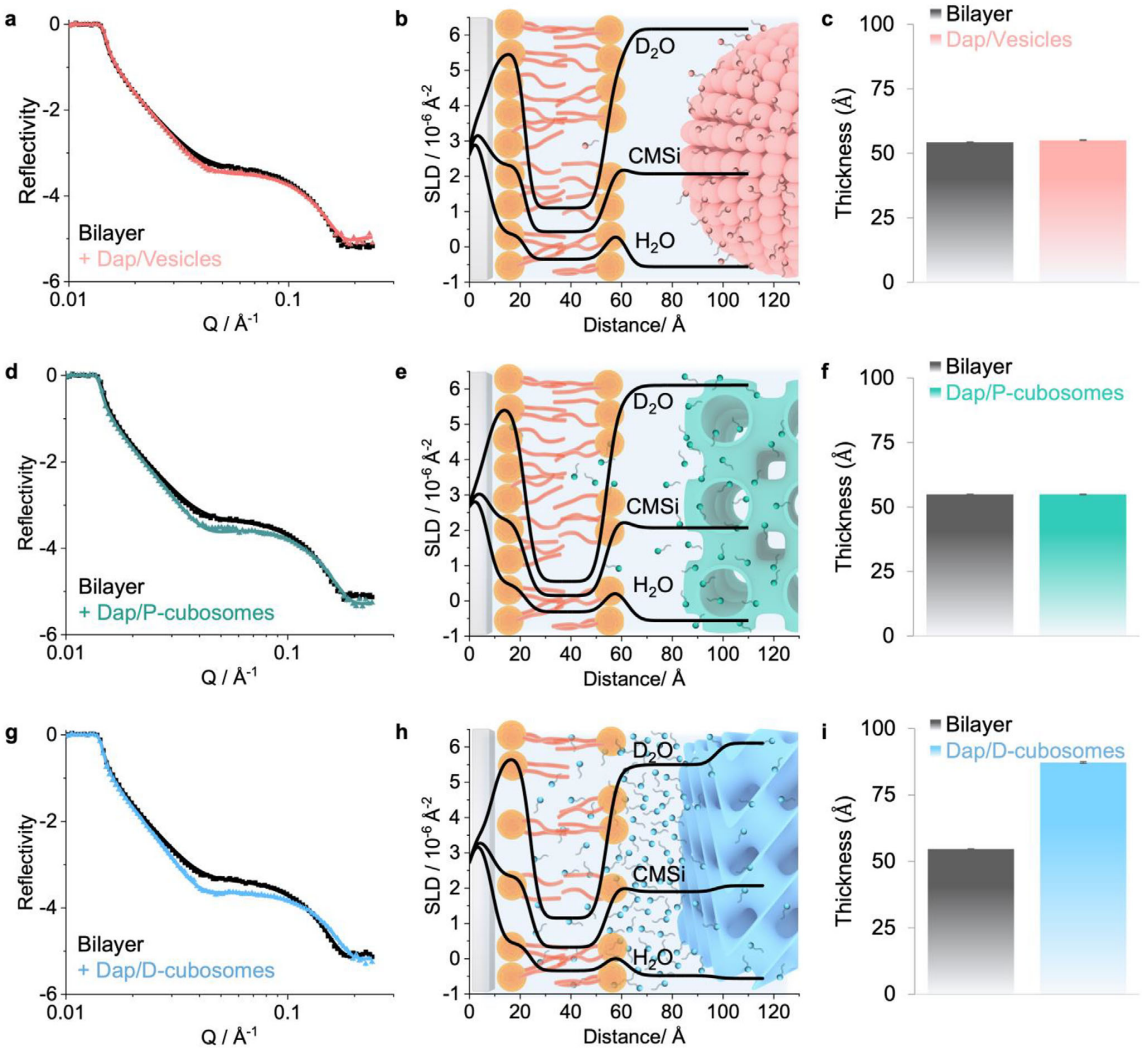
Neutron reflectometry analysis of daptomycin/LCNP interactions with *S. aureus* membrane models. (a) Neutron reflectivity profiles for the untreated bilayer (black curve) and post‐treatment with daptomycin/vesicles (red curve) in D_2_O, with enlarged sections highlighted on a yellow background. (b) Corresponding scattering length density (SLD) profiles with a background showing a visual representation of the post‐treatment bilayer structure. (c) Bilayer coverage comparisons before (black bar) and after (red bar) treatment. (d) Neutron reflectivity profiles for the bilayer treated with daptomycin/P‐cubosomes (green curve) in D_2_O. (e) SLD profiles post‐P‐cubosome treatment. **f** Bilayer coverage comparison before and after P‐cubosome treatment, shown as black and green bars, respectively. (g) Reflectivity profiles after introducing daptomycin/D‐cubosomes (blue curve) in D_2_O, with enlarged detail sections. (h) SLD profiles post‐D‐cubosome treatment, including an inset of the modified bilayer. (i) Bilayer coverage comparison before and after D‐cubosome treatment, represented by black and blue bars. All data are expressed as median ± SD (indicated by error bars), based on values obtained from three isotopic contrasts, i.e., D_2_O, CMSi (contrast‐matched silicon), and H_2_O. The data and fits for the CMSi and H_2_O contrasts corresponding to Figure 6b,e,h are shown in Figures S15–S17.

To differentiate interactions among nanostructures, three isotopic contrast solutions (D_2_O, H_2_O, and CMSi) were employed (Table S7). We then quantified interfacial changes for daptomycin/vesicles (Figure 6a–c, Figure S15 and Table S8), daptomycin/P‐cubosomes (Figure 6d–f, Figure S16 and Table S9), and daptomycin/D‐cubosomes (Figure 6g–i, Figure S17 and Table S10). The initial membrane bilayer exhibited a well‑defined Kiessig fringe at Q ∼ 0.04 Å^−1^ and a fitted thickness of 55.1 ± 0.1 Å, confirming successful bilayer formation (Figure 6a–c and Figure S15 and Table S8) [34]. Following treatment with daptomycin/vesicles, a decrease in reflectivity indicated perturbation of the bilayer (Figure 6a, red curve). Model fitting returned a 9.5% reduction in the bilayer volume fraction with no measurable change in thickness (Figure 6b,c, Table S8). The limited structural perturbation correlates with the minimal antimicrobial effects observed in microscopy studies and in vitro tests (Figures 2, 3, 4, Figures S6 and S7), indicating that lamellar vesicles do not substantially disrupt the membrane.

In contrast, daptomycin/P‐cubosomes led to a greater reduction in reflectivity at the same Q‐value (Figure 6d, green curve, Figure S16). Quantitative analysis revealed a 17.8% loss of lipid from the bilayer, consistent with partial lipid extraction rather than complete bilayer removal (Figure 6e,f, Table S9). The intermediate disruption aligns with TEM observations, where P‐cubosomes associate closely with the bacterial envelope and induce localized membrane deformation (Figure 4f), consistent with moderate curvature‐driven membrane interaction.

The most pronounced effect was observed with daptomycin/D‐cubosome. Reflectivity decreased across the entire Q‑range and the Kiessig fringe was largely suppressed (Figure 6g, blue curve). Model fitting showed a 36% reduction in lipid volume fraction, consistent with extensive lipid removal and increased water penetration into the bilayer (Figure 6h,i, Figure S17 and Table S10). Further analysis identified a D‐cubosome‐associated layer, measuring 33.5 ± 0.9 Å, deposited atop the membrane (Figure 6i, Table S10).

These findings are consistent with increased membrane interaction for highly curved LCNPs, in agreement with molecular simulations suggesting that nanoparticles with higher curvature can induce lipid redistribution and membrane deformation [52, 53]. The pronounced membrane disturbances observed in TEM images (Figure 4g) further support this trend, indicating that D‐cubosomes exhibit the strongest membrane interaction among the tested formulations. Such interactions are consistent with disruption of lipid packing and increased membrane permeability, which correlate with the enhanced antimicrobial activity (Figures 2, 3, and Tables S4–S6). SEM observations further reveal distinct membrane deformations following upon D‐cubosome treatment (Figure 4a), consistent with curvature‐enhanced membrane disruption.

## 3 Discussion

The present study differs from previous LCNP‐based antibacterial systems by isolating curvature as the primary structural variable within a matched nanoparticle platform. While prior studies have demonstrated membrane interaction, fusion, and delivery effects, these outcomes are typically confounded by concurrent variations in payload, lipid composition, or environmental conditions. By directly comparing lamellar vesicles, P‐cubosomes, and D‐cubosomes derived from the same lipid system and evaluated under identical assay conditions, we identify a consistent curvature‐dependent hierarchy in membrane disruption and antibacterial potentiation. These findings indicate that nanoparticle curvature itself plays a central role in governing membrane activity, independent of compositional differences.

To understand the underlying mechanisms driving structural transformations of LCNPs, we first consider the environmental factors that regulate lipid organization. Ionic strength directly influences lipid packing and bilayer curvature, both of which are critical determinants of LCNP phase behavior [23, 26, 27, 54]. This relationship becomes particularly evident with the addition of PBS, which alters electrostatic interactions between lipid headgroups, leading to measurable changes in curvature and particle morphology [26]. These transformations can be quantitatively described in terms of the surface‐average Gaussian curvature (*K*), a parameter linked to the packing properties of lipid aggregates. Gaussian curvature (*K*) is given by [29]: *K* = [3/(2*l_c_* ^2^)] * (1 – *P*), where *P* = *ν* / (*a_0_ · l_c_*) describes lipid packing [28], *v* represents the molecular volume of the fluid hydrocarbon chain(s), *a_0_* denotes the interfacial area per molecule at the polar–non‐polar interface, and *l_c_* indicates the critical tail length. When PBS is introduced, electrostatic screening reduces the interfacial area (*a_0_*)​, thereby increasing *P* and shifting the system toward phases with more negative curvature (Table S3).

**FIGURE 7:**
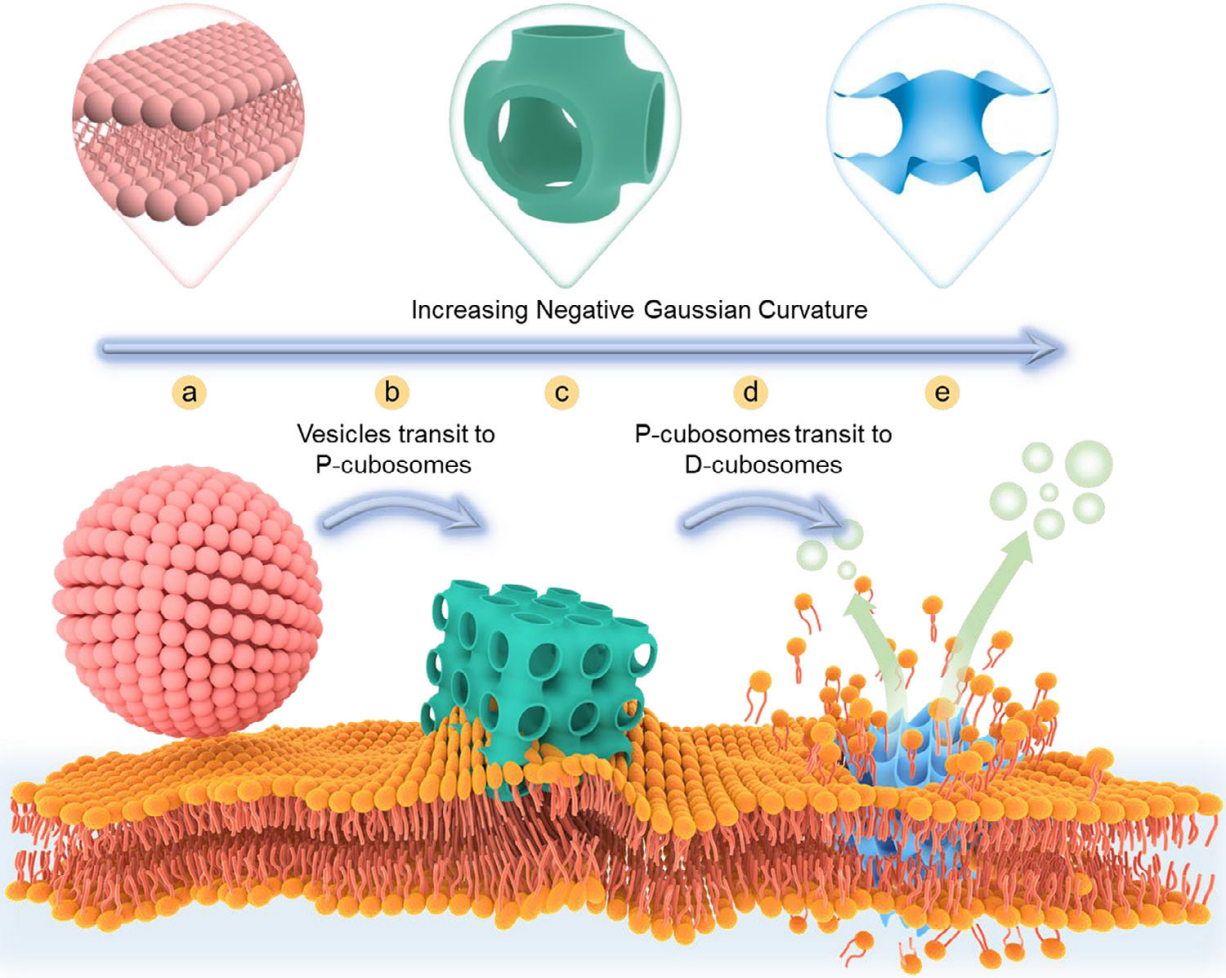
Interaction mechanism of LCNPs with the *S. aureus* membrane following daptomycin‑induced bilayer destabilization. The LCNP unit cells were drawn individually to clearly illustrate their increasing negative Gaussian curvature. (a) Co‐assembly of 15 wt.% DPPS with lyotropic phytantriol forms lamellar vesicles. These vesicles remain near the bacterial membrane but exhibit weak antimicrobial activity due to ineffective binding. (b) Vesicles transition into the P‐cubosomes in 0.1X PBS. (c) P‐cubosomes, characterized by relatively high Gaussian curvature, attach to the membrane surface and partially penetrate the bilayer without causing significant disruption. (d) P‐cubosomes transform into D‐cubosomes in 1X PBS. (e) D‐cubosomes, with the highest Gaussian curvature, adhere to and infiltrate the bilayer through membrane fusion, leading to membrane rupture, content leakage, and bacterial cell damage.

This structural transition provides a basis for the observed differences in antibacterial potentiation. In their lamellar vesicle form, LCNPs exhibit relatively flat bilayers with minimal curvature, resulting in weak interaction with bacterial membranes and reduced antimicrobial activity (Figure 7a). This observation is consistent with the reduced membrane interaction measured in Figures 2 and 3, as their flatter surface provides insufficient contact area for significant mechanical interaction, preventing vesicles from compromising bacterial membrane integrity [55]. Upon transitioning into P‐cubosomes (Figure 7b), the increased curvature of the intermediate cubic phase enhances the interaction potential with bacterial membranes (Figure 7c). The final transition into D‐cubosomes is associated with more negative Gaussian curvature, which further increases membrane‐disrupting potential compared with vesicles and P‐cubosomes (Figure 7d,e). This enhanced activity can be understood through Helfrich's curvature‐elastic energy model, which relates the energy stored in a membrane to its curvature [10]. According to this model: $E=\int \left[2k{\left(H-H_{0}\right)}^{2}+\bar{k}K\right]\mathrm{dA}$, where the elastic energy *E* increases with deviations from spontaneous curvature (*H_0_*) and is further influenced by Gaussian curvature. In the case of D‐cubosomes, the sharp negative Gaussian curvature (*K* < 0, Table S3) and deviation from *H_0_* generate localized stress at the nanoparticle‐membrane interface. Such stress can weaken lipid packing, facilitate lipid rearrangement, and increase membrane permeability [56], consistent with the enhanced membrane perturbation observed across microscopy (Figures 2, 3, 4). Although all whole‐cell experiments (Figures 2, 3, 4) retain the native peptidoglycan‐containing envelope, they do not by themselves resolve which envelope component contributes most directly to the curvature‐dependent membrane damage. The peptidoglycan‐labeling experiment helps place this mechanism in context (Figure S14). Rather than indicating strong LCNP accumulation within the peptidoglycan layer, the imaging supports the view that the cell wall mainly provides the native outer barrier through which LCNP–cell interactions occur. Therefore, neutron reflectometry was used as a complementary reductionist approach to isolate the lipid‐membrane remodeling step (Figure 6). Although this model does not reproduce the full Gram‐positive envelope, it avoids the additional structural heterogeneity and scattering complexity of peptidoglycan, enabling quantitative assessment of lipid loss, hydration changes and bilayer disruption. The same vesicles < P‐cubosomes < D‐cubosomes trend in intact‐cell assays and in the lipid‐membrane model supports membrane remodeling as a key downstream mechanism, consistent with the membrane‐targeting activity of daptomycin [57]. This model may therefore provide a useful framework for understanding curvature‐mediated interactions of membrane‐active nanomaterials in future studies.

However, although post‐mixing SAXS, DLS and zeta‐potential measurements (Figure S2, Table S2) support retention of the assigned LCNP phases and do not indicate major colloidal restructuring after co‐administration with daptomycin, these measurements do not exclude transient molecular‐scale association or partitioning of daptomycin into the lipid phase. The proposed potentiation mechanism therefore does not depend on stable daptomycin loading or persistent partitioning into the cubic phase. Instead, because daptomycin was added as a free drug at a constant concentration and only the LCNP internal structure was varied, the data support a co‐administration mechanism in which daptomycin increases membrane susceptibility while retained LCNP curvature governs the extent of membrane remodeling and disruption.

Meanwhile, several limitations should be considered when interpreting the translational significance of this work. Although SAXS confirmed structural retention in the final in vitro assay medium with or without daptomycin (Figures S2 and S3), structural evolution in vivo cannot be excluded because serum proteins, lipoproteins, and endogenous lipases may remodel LCNPs before they reach infected tissues. The in vivo experiment therefore demonstrates a proof‐of‐concept efficacy trend under the tested co‐administration conditions rather than therapeutic validation. Future translational development will require serum or plasma stability studies, quantitative assessment of daptomycin association or loading, pharmacokinetic/pharmacodynamic analysis, biodistribution, dose optimization, repeated‐dose and long‐term safety assessment, immunogenicity evaluation, and comparison of co‐administered versus drug‐loaded formulations. These studies will be needed to determine whether curvature‐programmed LCNPs can be advanced from a mechanistic potentiation platform to a clinically relevant antimicrobial strategy.

In conclusion, this study demonstrates that LCNP curvature regulates membrane interaction and disruption in a controlled antibacterial system. The ability to transition between lamellar and cubic phases provides a means to systematically tune curvature and thereby modulate antimicrobial activity. These findings establish curvature as a design parameter for membrane‐active nanomaterials, offering a framework for developing LCNP‐based antimicrobial strategies against multidrug‐resistant pathogens such as MRSA.

## 4 Materials and Methods

### 4.1 Preparation of LCNPs and Sequential Phase‐Transition Workflow

15 wt.% DPPS and 85 wt.% phytantriol were used to create vesicles, while P‐cubosomes and D‐cubosomes were formed by dispersing the same formulation in 0.1X and 1X phosphate‐buffered saline (PBS), respectively. The LCNPs were created by mixing phytantriol, DPPS, and a 10% Pluronic F127 chloroform solution. This mixture was dried under nitrogen gas, followed by overnight vacuum drying to form a soft lipid gel. The gel was probe‐ultrasonicated (Qsonica, 125 W, 20 kHz; 50% amplitude, 5 min, 5 s on/5 s off) into the selected dispersant to generate the nanoparticles. The workflow was sequential: (i) the gel was first dispersed in Milli‐Q water to produce a vesicle dispersion; (ii) an aliquot of this vesicle stock was then dispersed in 0.1× PBS (same sonication protocol) to generate P‐cubosomes; (iii) the P‐cubosome dispersion was subsequently dispersed in 1× PBS (same sonication protocol) to generate D‐cubosomes. The final LCNP concentration in each case was calculated from the starting amounts of phytantriol, DPPS, and F127. Only after SAXS/cryo‐TEM confirmation of the internal phase were all three dispersions diluted into the same culture medium used for physicochemical and biological measurements.

### 4.2 Dynamic Light Scattering

The particle size, polydispersity index, and zeta potential of the LCNPs were measured using a Malvern Zetasizer Nano ZS (Zetasizer Software V6.28). Samples were diluted to 0.1 mg/mL in either Milli‐Q water or 1X PBS and loaded into DTS 1070 folded capillary cells. The comparative DLS and zeta‐potential measurements were performed after all LCNPs were transferred into the same final measurement medium. Measurements were conducted at 37°C using Dynamic Light Scattering (DLS) with a backscattering angle of 173°, with each sample measured in triplicate for 60 s per reading.

### 4.3 Small Angle X‐Ray Scattering (SAXS)

SAXS analysis was performed at 25°C using a Bruker N8 Horizon instrument with Diffrac. Suite V7.3.1 software (Monash X‐ray Platform, Monash University, Australia). The system utilized a Cu‐anode (wavelength λ = 1.5406 Å) for Kα radiation, operating at 50 keV and 1000 µA, covering a Q‐range from 0.007 to 0.387 Å^−1^. The scattering vector (Q) is calculated using [4π sin(θ/2)]/λ, where θ is the scattering angle. LCNPs (100 mg/mL) were loaded into a quartz capillary, with Milli‐Q water as the background and glassy carbon as the reference. Measurements were performed under vacuum, and a VÅNTEC‐500 Detector captured the 2D scattering pattern. The data were subsequently integrated into a 1D function and background‐subtracted using DIFFRAC.SAXS software (Monash X‐ray Platform, Monash University, Australia). Calibration was done using a silver behenate standard (d‐spacing = 58.38 Å). The unit cell parameter (a) was determined using the formula: $a=d_{hkl}\;\sqrt{\left(h^{2}+k^{2}+l^{2}\right)}$, where the lattice spacing *d*_hkl_ = 2π/*Q*, h, k, and l are miller indices. The diameter of the water channel in a cubic phase is determined by calculating the radius of the water channel (*r_w_*), which is estimated using the lattice parameter based on minimal surfaces [23]: for *Pn3m r_w_* = 0.391*a*—*L*, for *Im3m r_w_* = 0.305*a*—*L*, where *L* is the lipid chain length (ca. 1.4 nm for phytantriol) [58]. The Gauss‐Bonnet theorem establishes a relationship between the Gaussian curvature <*K>* and the lattice parameter via <*K>* = 2π*χ*/(*σ_0_a* ^2^), where *χ* and *σ* are topological constants (*σ_0_* = 1.919 and *χ* = − 2 for *Pn3m* phase; *σ_0_* = 2.345 and *χ* = − 4 for *Im3m* phase) [23, 24].

### 4.4 Cryogenic Transmission Electron Microscopy (Cryo‐TEM)

Cryo‐TEM samples were prepared using a humidity‐controlled vitrification system, set to 80% humidity and 22°C. Hydrophilic 300‐mesh copper grids with Lacey carbon film (ProSciTech #GSCU300FL‐50, QLD, Australia) were used. A 3 µL aliquot of each sample was applied to the grids, followed by a 5 s adsorption and 2 s blotting using Whatman 541 filter paper. The grids were then rapidly vitrified by plunging them into liquid ethane cooled by liquid nitrogen and subsequently stored in liquid nitrogen. Observations were performed using a Gatan 626 cryoholder (Gatan, Pleasanton, CA, USA) with a Tecnai 12 Transmission Electron Microscope (FEI, Eindhoven, The Netherlands) operating at 120 kV. Low‐dose procedures were employed, with an electron dose of 8–10 electrons/Å^2^. Images were captured using an FEI Eagle 4k x 4k CCD camera and processed with AnalySIS v3.2 software (Olympus).

### 4.5 Combination‐Treatment Workflow (Co‐Administration)

For all combination‐treatment assays, free daptomycin (0.25 µg/mL) and the indicated blank LCNP formulation (64 µg/mL) were added independently and directly into the final assay medium. No daptomycin‐loading or encapsulation step was performed.

### 4.6 Membrane Interaction

MRSA in the logarithmic phase was diluted to a final concentration of ∼10^6^ CFU/mL in saline. Rhodamine B‐labeled LCNPs were added to the bacterial suspensions, and the mixtures were incubated for 4 h at 37°C with shaking (200 rpm). Following treatment, bacterial suspensions (1 mL) were incubated with CellBrite Fix 488 membrane stain for 15 min at 37°C to prepare for imaging. Confocal images were acquired using an Olympus FV3000 microscope equipped with a cooled GaAsP photomultiplier detector and a ×100 objective (NA 1.40, oil immersion) lens at Monash University, Australia.

### 4.7 Outer Membrane Permeability Assay

To evaluate outer membrane permeability, the 1‐N‐phenylnaphthylamine (NPN) uptake assay was conducted [30, 31, 32]. NPN is a hydrophobic fluorescent probe that exhibits minimal fluorescence in aqueous environments but displays strong fluorescence when it reaches the hydrophobic core of disrupted membranes. For this assay, MRSA A8819 cells were prepared at a concentration of 2 × 10^6^ cells mL^−1^ in 5 mmol L^−1^ HEPES buffer (pH 7.2). NPN was added to a final concentration of 20 µM, and the samples were incubated for 60 min with LCNPs at varying concentrations (16–64 µg mL^−1^). Fluorescence emission intensity (λ_exc_ = 340 nm, λ_em_ = 405 nm) was recorded using a Multimode Microplate Reader VICTOR Nivo (PerkinElmer). The microtiter plate wells contained the following components: (1) 100 µL of bacterial suspension in HEPES buffer, (2) 50 µL of 80 mmol L^−1^ NPN solution in the same buffer, and (3) LCNPs diluted in 50 µL of buffer. Fluorescence readings were corrected for background by subtracting the values obtained in the absence of NPN. The results were expressed as NPN uptake factors, calculated as the ratio of the background‐corrected fluorescence of the bacterial suspension to that of the buffer.

### 4.8 Minimum Inhibitory Concentration (MIC)

Colony‐forming unit (CFU) measurement was used to determine the viable bacterial count in each sample. Two‐fold dilutions of the antimicrobial agent were prepared in Mueller–Hinton Broth (MHB containing 50 mg/L Ca^2^^+^ and 10–12.5 mg/L Mg^2^^+^). A 96‐well microtiter plate was then loaded with 100 µL of these antimicrobial dilutions. Bacterial suspensions of *S. aureus* were adjusted to approximately 10^8^ CFU/mL and diluted 1:100 in MHB to achieve a concentration of 10^6^ CFU/mL. Within 15 min, 100 µL of these diluted bacterial suspensions were vortex‐mixed and added to the wells, resulting in a final inoculum concentration of approximately 5 × 10^5^ CFU/mL. The minimum inhibitory concentration (MIC) was determined after a 16–20 h incubation at 37°C. All procedures were performed in triplicate (*n* = 3) for consistency.

### 4.9 Fractional Inhibitory Concentration Index (FICI)

Checkerboard micro‐dilution assays were performed immediately after the MIC measurements to quantify the interaction between daptomycin and each LCNP formulation. In sterile 96‐well plates, two‐fold serial dilutions of daptomycin (horizontal axis) were crossed with two‐fold serial dilutions of vesicles, P‐cubosomes, or D‐cubosomes (vertical axis) in MHB containing 50 mg/L Ca^2^^+^. Each well therefore contained a unique combination of the two agents plus ∼5 × 10^5^ CFU/mL *S. aureus* inoculum in a final volume of 200 µL. After 18 h incubation at 37°C, the lowest concentration combination that inhibited visible growth was recorded. FICI = [daptomycin]_combo_/MIC_daptomycin_ + [LCNP]_combo_/MIC_LCNP_, where [agent]_combo_ is the concentration present in the first growth‐inhibitory well. FICI values were interpreted as follows: ≤ 0.5, synergy; 0.5–4.0, indifference; > 4.0, antagonism. All checkerboard experiments were run in triplicate.

### 4.10 Confocal Microscopy

For the live/dead assay, MRSA A8819 suspensions (∼10^6^ CFU/mL, 5 mL) were treated with daptomycin, LCNPs, or their combination, and incubated at 37°C for 4 h with shaking at 200 rpm. The mixtures were then washed twice by centrifugation at 10 000× g for 10 min in 1× PBS. After staining with SYTO 9 and propidium iodide for 15 min in darkness, images were captured using an Olympus FV3000 confocal microscope equipped with a cooled GaAsP photomultiplier detector.

For peptidoglycan labeling, overnight MRSA A8819 cultures were diluted into fresh medium and grown to mid‐log phase at 37°C with shaking. Cells were incubated with 7‐hydroxycoumarin carbonyl amino‐D‐alanine (HADA, 250 µM, Sigma) for 30 min at 37°C to label newly synthesized peptidoglycan, then washed twice with 1× PBS to remove unincorporated dye. HADA‐labeled cells were then treated with Rhodamine B‐labeled LCNPs (64 µg/mL) under the same treatment conditions described above. After incubation, samples were washed twice with 1× PBS and imaged by confocal microscopy using separate channels for HADA and Rhodamine B. Overlap between the peptidoglycan and LCNP signals was assessed from merged fluorescence images.

### 4.11 Quantification of Bacterial Viability

Bacterial viability was assessed using the LIVE/DEAD BacLight Bacterial Viability Kit (Thermo Fisher Scientific) with slight modifications for fluorescence detection using a microplate reader. *MRSA* cultures were grown to the logarithmic phase, washed with saline, and adjusted to ∼2 × 10^8^ CFU/mL. The bacterial suspensions were treated with daptomycin, LCNPs, and their combinations. After treatment, 0.5 µL of SYTO 9 and propidium iodide (PI) dye mixture was added to 100 µL of bacterial suspension, followed by direct incubation without a washing step to avoid cell loss. For the standard curve, live and dead bacterial suspensions were generated by incubating live *MRSA* with saline or 70% isopropyl alcohol (for dead cells) for 1 h. Serial dilutions of live‐to‐dead cells were prepared (0:100, 10:90, 50:50, 90:10, 100:0). Each treatment (daptomycin, LCNPs, and their combinations) was stained and measured by fluorescence spectroscopy to obtain the background spectrum. Following incubation in the dark for 15 min, fluorescence was measured using a microplate reader with excitation at 485 nm. SYTO 9 fluorescence was detected at 530 nm (green, live cells), and PI fluorescence was detected at 630 nm (red, dead cells). The background spectrum of each treatment was subtracted from the sample spectra, and the average spectrum from triplicate measurements was used for regression model calculation. Bacterial viability was determined as the percentage of live cells using the SYTO 9/PI ratio, and the standard curve was generated by plotting the green/red fluorescence ratio against live cell percentages.

### 4.12 Membrane Permeability to Detect Oxidative Stress

2',7'‐Dichlorodihydrofluorescein diacetate (DCFH‐DA) was used as a fluorescent probe. Bacterial cultures were grown to an optical density of McFarland 0.5, then centrifuged at 5000× g for 5 min and washed twice with saline. The resulting pellet was resuspended in saline to a final concentration of 10^6^ CFU/mL, after which DCFH‐DA was added at a final concentration of 5 µM. The mixture was incubated in the dark at 37°C for 45 min with gentle shaking at 100 rpm. Following this incubation, the appropriate treatments were applied, and the samples were incubated again at 37°C with shaking at 200 rpm. Control samples included untreated cells (negative control), cells treated with only DCFH‐DA (positive control), experimental samples with DCFH‐DA and treatment, and wells containing saline and DCFH‐DA to measure background fluorescence. Fluorescence intensity was measured every 15 min for 24 h using a microplate reader with an excitation wavelength of 485 nm and an emission wavelength of 530 nm. Fluorescence readings were normalized to the negative control and compared with the positive control to assess membrane permeability. All steps involving DCFH‐DA were performed under low light conditions to prevent photo‐oxidation, and all measurements were conducted in triplicate for accuracy.

### 4.13 Scanning Electron Microscopy (SEM)

The morphology of MRSA A8819 (10^6^ CFU/mL), either untreated or treated with daptomycin, LCNPs, or a combination of both, was examined using scanning electron microscopy (SEM) after 4 h of exposure at 37°C. After treatment, the bacterial suspensions were centrifuged at 10 000× g for 10 min at 4°C, and the resulting pellets were fixed overnight in Karnovsky fixative at 4°C. Following fixation, the samples were washed three times with 0.1 M sodium cacodylate buffer, then subjected to secondary fixation using osmium tetroxide and potassium ferricyanide. The cells were washed three additional times with Milli‐Q water, placed on poly‐L‐lysine‐coated coverslips, and dehydrated through an ethanol gradient (30%, 50%, 70%, 90%, and 100%, for 10 min at each step). The samples were then dried using a Bal‐Tech CPD 030 critical point dryer. Coverslips were mounted onto aluminum SEM stubs, gold‐coated using a Bal‐Tec SCD 005 sputter coater, and imaged using a FEI Nova NanoSEM 450 FEGSEM.

### 4.14 Transmission Electron Microscopy (TEM)

Bacteria were initially centrifuged at 5000× g for 5 min to obtain a pellet, which was then fixed overnight at 4°C using Karnovsky fixative. Afterward, the pellet was centrifuged again at 5000× g for 5 min and washed twice with 0.1 M cacodylate buffer (pH 7.4). The cells, along with the LCNPs, were stained with 2% uranyl acetate solution. Following two additional washes with deionized water, the samples were mounted on carbon‐coated copper grids and imaged at 80 keV using a JEOL JEM‐1400PLUS TEM, equipped with a high‐sensitivity bottom‐mount CMOS “Flash” camera.

### 4.15 Cell Viability Test

Human embryonic kidney (HEK‑293T; Sigma #12022001; RRID: CVCL_0063) cells were seeded into 96‑well, tissue‑culture–treated plates at 5 × 10^3^ cells per well (100 µL complete medium) and incubated overnight at 37°C, 5 % CO_2_. Cells were then exposed for 24 h to medium containing graded concentrations of daptomycin, LCNPs or their combination. Without removing the treatment medium, 10 µL of MTT labeling reagent (5 mg/mL stock, final assay concentration 0.5 mg/mL) were added to each well, and plates were returned to the incubator for 4 h. Subsequently, 100 µL of ready‑to‑use solubilization buffer (10 % SDS in 0.01 M HCl) was added directly to each well, and the plates were left overnight at 37°C to ensure complete dissolution of the formazan crystals. Absorbance was recorded at 570 nm (reference > 650 nm) using a VICTOR Nivo plate reader, and cell viability was calculated relative to untreated controls.

### 4.16 In Vivo Efficacy

*In vivo* activity was evaluated in a murine *S. aureus* bacteraemia model (protocol number: 32532). All procedures were approved by the Monash University Animal Ethics Committee and performed in accordance with the Australian Code for the Care and Use of Animals for Scientific Purposes (8th Edition, 2013). Male and female BALB/c mice (8 weeks old, body‑weight 18–25 g) were obtained from Monash Animal Services and supplied with food and water ad libitum. Eight experimental groups (*n* = 5 mice per group) were studied: saline control, daptomycin, vesicles, P‑cubosomes, D‑cubosomes, daptomycin/vesicles, daptomycin/P‑cubosomes and daptomycin/D‑cubosomes. Animals were randomly assigned to treatment. A single colony of *S. aureus* (clinical isolate, methicillin susceptible) was cultured overnight (Brain Heart Infusion, 37°C, 200 r.p.m.). Cells were harvested (4000 × g, 10 min, 4 °C), washed twice and resuspended in sterile PBS. The suspension was adjusted to 1 × 10^8^ CFU/mL. 100 µL (≈1 × 10^7 ^CFU) was injected intravenously via the lateral tail vein to establish bloodstream infection. The actual inoculum was confirmed retrospectively by plating on BHI agar. Daptomycin stock was prepared in sterile saline at 1 mg/mL (injection volume  =  10 µL/g), mice received 10 mg/kg to mimic sub‐optimal therapeutic daptomycin concentration in vivo. LCNP dispersions were freshly prepared at 4.56 mg/mL, affording a dose of 45.6 mg/kg. Combination groups received daptomycin (1 mg/mL) and the relevant LCNP (4.56 mg/mL) in the same syringe immediately before administration. All treatments were delivered intraperitoneally 1 h post‑infection. Control animals received an equivalent volume of sterile saline. 24 h after infection, mice were euthanized with isoflurane. Kidneys, livers and spleens were aseptically excised, weighed, placed in pre‑weighed tubes containing 1 mL PBS, and homogenized at 20 000 rpm. for 30 s. Homogenates were serially diluted in 0.85 % saline, plated on BHI agar and incubated at 37°C for 18 h before colony enumeration. Bacterial burden was expressed as log10 CFU/g and reported as mean ± SEM for each group. Data were analyzed in Microsoft 365 (version 2105) and GraphPad Prism (version 9.0.1). Statistical analysis of in vivo bacterial‐burden data is described in the Statistical analysis subsection.

### 4.17 *S. aureus* Membrane Bilayer Formation for Neutron Reflectometry [34]

A model membrane was deposited onto a SiO_2_ surface using a custom Langmuir–Blodgett trough (Nima Technology, UK), following established Langmuir–Blodgett (LB) and Langmuir–Schaefer (LS) techniques [21]. A symmetric membrane bilayer, mimicking the composition of MRSA A8819, was constructed from a lipid mixture of synthetic PG (18:1), CL (18:1), and L‐PG (18:1) in a molar ratio of 69:12:19. The lipids, dissolved in chloroform, were spread onto a 5 mM CaCl_2_ water surface at 15°C. The bilayer was transferred onto the SiO_2_ surface in two steps: the inner leaflet was applied using LB deposition, and the outer leaflet was completed using the LS technique, both at a surface pressure ∼30 mN/m. The silicon wafer holding the bilayer was mounted in an aluminum holder with a silicon backing plate, equipped with inlet and outlet tubes to facilitate HEPES buffer exchange (5 mM CaCl_2_, 150 mM NaCl, 10 mM HEPES, pH 7.4).

### 4.18 Neutron Reflectometry Measurement [21]

The analysis was carried out on the Spatz and Platypus time‐of‐flight neutron reflectometers at the 20MW OPAL research reactor in Sydney, Australia, employing a cold neutron spectrum ranging from 2.8 to 18 Å. Spatz employs a horizontal scattering set‐up whilst Platypus has a vertical scattering setup [59, 60]. For both instrument the following experimental set‐up was used: neutron reflections from the sample over an illuminated footprint of 55 mm along the beam were captured at two angles of incidence (0.85° for 300 s and 3.5° for 3600 s), covering a momentum transfer (Q) range from 0.01 to 0.27 Å^−1^, where Q = 4π sin(θ)/λ, with θ as the incidence angle and λ as the wavelength. An instrument resolution of ΔQ/Q of ∼8% was used for Platypus, and ∼5% was used on Spatz.

The symmetric membrane bilayer's properties were examined using three isotopic contrasts for scattering length density (SLD): D_2_O (99.9%, ρ = 6.35 × 10^−6^ Å^−2^), CMSi (contrast‐matched silicon, 38% D_2_O: 62% H_2_O v/v; ρ = 2.07 × 10^−6^ Å^−2^) and H_2_O (ρ = −0.56 × 10^−6^ Å^−2^). A 7 mL HEPES buffer solution (pH/D 7.4) was circulated through the sample at 1.0 mL/min using a pump (Knauer GmbH, Berlin, Germany) for contrast exchange. The bilayer's initial characterization in buffered contrasts confirmed its formation. It was then exposed to 2 µg/mL daptomycin for 4 h, followed by a D2O buffer wash to remove non‐specific bindings. Post‐treatment, the bilayer was re‐characterized under all three contrasts. Subsequently, a second treatment with 64 µg/mL LCNPs was applied to study the combinational treatment effects on the MRSA model membrane.

Data analysis was streamlined using the refnx data reduction routine [61]. This routine adjusts for detector efficiency, converts time‐of‐flight data to wavelength for Q calculation, re‐bins data to match instrument resolution, and merges datasets from two incidence angles at their overlap to form a complete reflectivity profile. It also normalizes the data so that the critical edge corresponds to a reflectivity of one. The final reflectivity data is expressed in terms of momentum transfer.

### 4.19 Neutron Reflectometry Data Analysis

The neutron profiles were analyzed using refnx software [61], complemented by Markov‐Chain Monte Carlo methods to establish 95% confidence intervals [62]. Briefly, the bilayer was segmented into sublayers characterized by thickness, scattering length density (SLD) (Table S7), and roughness. Volume fractions of each component in these sublayers were calculated by fitting the same layer under different isotopic contrasts simultaneously. For layers comprising a chemical species s and water w, the SLD is expressed as *ρ_layer_* = *φρ_s_* + (1‐ *φ*) *ρ_w_*, where *ρ_s_* and *ρ_w_* are the SLD of the two components, respectively, and *φ* is the volume fraction of chemical species *s* in the layer. Considering the higher hydration in lipid headgroup regions due to their hydrophilicity, volume fractions were primarily determined by the lipid tail region's combined volume fractions [46].

### 4.20 Statistical Analysis

Statistical analyses were performed using GraphPad Prism version 9.0.1. Data are presented as mean ± SD for in vitro assays and physicochemical measurements, unless otherwise stated. In vivo bacterial burdens are presented as mean ± SEM of log_10_ CFU/g. Sample sizes are indicated in the corresponding Methods subsections and figure legends. For comparisons among multiple in vitro treatment groups, one‐way ANOVA followed by Tukey's multiple‐comparisons test was used. For in vivo bacterial‐burden comparisons, the Kruskal–Wallis test followed by Dunn's multiple‐comparisons test was used. *P* < 0.05 was considered statistically significant. Statistical significance is indicated as follows: ns, not significant; ^*^ *P* < 0.05; ^**^ *P* < 0.01; ^***^ *P* < 0.001; and ^****^ *P* < 0.0001.

## Author Contributions

X.L. conceived the project, prepared and characterized the structure of samples, carried out all experiments, analysed all the experimental data, and wrote the manuscript with contributions from all the authors. S.W. performed all animal experiments. C. D. provided foundation support. X. K. assisted with animal experiments. A.P.L.B. provided technical support for neutron experiments. H.‐Y. H. revised the manuscript. J. ‐H. J. provided 3 bacterial strains from the A. Y. P. group. Y. W. and R. A. S. provided idea support. A. Y. P. and H.‐H.S. conceived the project, provided the foundation support, and modified the structure of the manuscript.

## Conflicts of Interest

The authors declare no conflicts of interest.

## Supporting information




**Supporting File**: advs76516‐sup‐0001‐SuppMat.docx.

## Data Availability

The data that support the findings of this study are available in the supplementary material of this article.

## References

[1] T. Jesudason , “Maintaining a Robust Pipeline of Antibiotics,” The Lancet Infectious Diseases 23, no. 7 (2023): 791, 10.1016/S1473-3099(23)00384-5. 37392757

[2] T. R. Walsh , A. C. Gales , R. Laxminarayan , and P. C. Dodd , “Antimicrobial Resistance: Addressing a Global Threat to Humanity,” PLOS Medicine 20, no. 7 (2023): 1004264, 10.1371/journal.pmed.1004264. PMC10317217 37399216

[3] V. MA and U. Ts , “Overcoming Resistance,” Nature 586 (2020): S55, 10.1038/d41586-020-02886-1.

[4] J. M. V. Makabenta , A. Nabawy , C. H. Li , et al., “Nanomaterial‐Based Therapeutics for Antibiotic‐Resistant Bacterial Infections,” Nature Reviews Microbiology 19 (2020): 23–36, 10.1038/s41579-020-0420-1. 32814862 PMC8559572

[5] W. Gao and L. Zhang , “Nanomaterials Arising Amid Antibiotic Resistance,” Nature Reviews Microbiology 19, no. 1 (2021): 5–6, 10.1038/s41579-020-00469-5. PMC7538279 33024312

[6] F. A. Z. Sayed , N. G. Eissa , Y. Shen , D. A. Hunstad , K. L. Wooley , and M. Elsabahy , “Morphologic Design of Nanostructures for Enhanced Antimicrobial Activity,” Journal of Nanobiotechnology 20, no. 1 (2022): 536, 10.1186/s12951-022-01733-x. 36539809 PMC9768920

[7] M. Xie , M. Gao , Y. Yun , et al., “Antibacterial Nanomaterials: Mechanisms, Impacts on Antimicrobial Resistance and Design Principles,” Angewandte Chemie International Edition 62, no. 17 (2023): 202217345, 10.1002/anie.202217345. 36718001

[8] E. P. Ivanova , D. P. Linklater , M. Werner , et al., “The Multi‐Faceted Mechano‐Bactericidal Mechanism of Nanostructured Surfaces,” Proceedings of the National Academy of Sciences 117, no. 23 (2020): 12598–12605, 10.1073/pnas.191668011. PMC7293705 32457154

[9] D. P. Siegel , “The Gaussian Curvature Elastic Energy of Intermediates in Membrane Fusion,” Biophysical Journal 95, no. 11 (2008): 5200–5215, 10.1529/biophysj.108.140152. 18805927 PMC2586550

[10] W. Helfrich , “Elastic Properties of Lipid Bilayers: Theory and Possible Experiments,” Zeitschrift für Naturforschung c 28, no. 11–12 (1973): 693–703, 10.1515/znc-1973-11-1209. 4273690

[11] L. Zheng , S. R. Bandara , Z. Tan , and C. Leal , “Lipid Nanoparticle Topology Regulates Endosomal Escape and Delivery of RNA to the Cytoplasm,” Proceedings of the National Academy of Sciences 120, no. 27 (2023): 2301067120, 10.1073/pnas.2301067120. PMC10318962 37364130

[12] B. P. Dyett , H. Yu , J. Strachan , C. J. Drummond , and C. E. Conn , “Fusion Dynamics of Cubosome Nanocarriers with Model Cell Membranes,” Nature Communications 10, no. 1 (2019): 4492, 10.1038/s41467-019-12508-8. PMC6776645 31582802

[13] B. P. Dyett , H. Yu , S. Sarkar , J. B. Strachan , C. J. Drummond , and C. E. Conn , “Uptake Dynamics of Cubosome Nanocarriers at Bacterial Surfaces and the Routes for Cargo Internalization,” ACS Applied Materials & Interfaces 13, no. 45 (2021): 53530–53540, 10.1021/acsami.1c09909. 34726885

[14] A. Scheeder , M. Brockhoff , E. N. Ward , G. S. Kaminski Schierle , I. Mela , and C. F. Kaminski , “Molecular Mechanisms of Cationic Fusogenic Liposome Interactions with Bacterial Envelopes,” Journal of the American Chemical Society 145, no. 51 (2023): 28240–28250, 10.1021/jacs.3c11463. 38085801 PMC10755748

[15] B. P. Dyett , S. Sarkar , H. Yu , J. Strachan , C. J. Drummond , and C. E. Conn , “Overcoming Therapeutic Challenges of Antibiotic Delivery with Cubosome Lipid Nanocarriers,” ACS Applied Materials & Interfaces 16, no. 19 (2024): 24191–24205, 10.1021/acsami.4c00921. 38690584

[16] S. Subramaniam , P. Joyce , and C. A. Prestidge , “Liquid Crystalline Lipid Nanoparticles Improve the Antibacterial Activity of Tobramycin and Vancomycin Against Intracellular Pseudomonas Aeruginosa and Staphylococcus Aureus,” International Journal of Pharmaceutics 639 (2023): 122927, 10.1016/j.ijpharm.2023.122927. 37059243

[17] L. Boge , A. Umerska , N. Matougui , et al., “Cubosomes Post‐Loaded with Antimicrobial Peptides: Characterization, Bactericidal Effect and Proteolytic Stability,” International Journal of Pharmaceutics 526, no. 1–2 (2017): 400–412, 10.1016/j.ijpharm.2017.04.082. 28476579

[18] L. Boge , K. L. Browning , R. Nordström , et al., “Peptide‐Loaded Cubosomes Functioning as an Antimicrobial Unit against Escherichia coli,” ACS Applied Materials & Interfaces 11, no. 24 (2019): 21314–21322, 10.1021/acsami.9b01826. 31120236

[19] M. Gontsarik , M. T. Buhmann , A. Yaghmur , Q. Ren , K. Maniura‐Weber , and S. Salentinig , “Antimicrobial Peptide‐Driven Colloidal Transformations in Liquid‐Crystalline Nanocarriers,” The Journal of Physical Chemistry Letters 7, no. 17 (2016): 3482–3486, 10.1021/acs.jpclett.6b01622. 27541048

[20] L. Hong , M. Gontsarik , H. Amenitsch , and S. Salentinig , “Human Antimicrobial Peptide Triggered Colloidal Transformations in Bacteria Membrane Lipopolysaccharides,” Small 18, no. 5 (2022): 2104211, 10.1002/smll.202104211. 34825488

[21] X. Lai , M. L. Han , Y. Ding , et al., “A Polytherapy Based Approach to Combat Antimicrobial Resistance Using Cubosomes,” Nature Communications 13, no. 1 (2022): 1–12, 10.1038/s41467-022-28012-5. PMC8763928 35039508

[22] J. H. Jiang , C. X. Lim , X. Lai , et al., “Daptomycin‐Loaded Nanocarriers Facilitate Synergistic Killing of Methicillin‐Resistant Staphylococcus aureus via Lipid‐Mediated Interactions and Targeting,” The Journal of Infectious Diseases 233, no. 1 (2026): e22–e33, 10.1093/infdis/jiaf492. 41189495 PMC12811857

[23] M. Salim , W. F. N. Wan Iskandar , M. Patrick , N. I. Zahid , and R. Hashim , “Swelling of Bicontinuous Cubic Phases in Guerbet Glycolipid: Effects of Additives,” Langmuir 32, no. 22 (2016): 5552–5561, 10.1021/acs.langmuir.6b01007. 27183393

[24] R. H. Templer , J. M. Seddon , N. A. Warrender , et al., “Inverse Bicontinuous Cubic Phases in 2:1 Fatty Acid/Phosphatidylcholine Mixtures. The Effects of Chain Length, Hydration, and Temperature,” The Journal of Physical Chemistry B 102, no. 37 (1998): 7251–7261, 10.1021/jp972835a.

[25] J. Seddon and R. Templer , “Polymorphism of Lipid‐Water Systems,” Handbook of Biological Physics 1 (1995): 97–160, 10.1016/S1383-8121(06)80020-5.

[26] B. W. Muir , G. Zhen , P. Gunatillake , and P. G. Hartley , “Salt Induced Lamellar to Bicontinuous Cubic Phase Transitions in Cationic Nanoparticles,” The Journal of Physical Chemistry B 116, no. 11 (2012): 3551–3556, 10.1021/jp300239g. 22360659

[27] Q. Liu , Y.‐D. Dong , T. L. Hanley , and B. J. Boyd , “Sensitivity of Nanostructure in Charged Cubosomes to Phase Changes Triggered by Ionic Species in Solution,” Langmuir 29, no. 46 (2013): 14265–14273, 10.1021/la402426y. 24111826

[28] R. A. Khalil and A. A. Zarari , “Theoretical Estimation of the Critical Packing Parameter of Amphiphilic Self‐Assembled Aggregates,” Applied Surface Science 318 (2014): 85–89, 10.1016/j.apsusc.2014.01.046.

[29] A. I. I. Tyler , H. M. G. Barriga , E. S. Parsons , et al., “Electrostatic Swelling of Bicontinuous Cubic Lipid Phases,” Soft Matter 11, no. 16 (2015): 3279–3286, 10.1039/c5sm00311c. 25790335

[30] A. G. Elliott , J. X. Huang , S. Neve , et al., “An Amphipathic Peptide with Antibiotic Activity Against Multidrug‐Resistant Gram‐Negative Bacteria,” Nature Communications 11, no. 1 (2020): 3184, 10.1038/s41467-020-16950-x. PMC7311426 32576824

[31] T. Velkov , A. Gallardo‐Godoy , J. D. Swarbrick , et al., “Structure, Function, and Biosynthetic Origin of Octapeptin Antibiotics Active Against Extensively Drug‐Resistant Gram‐Negative Bacteria,” Cell Chemical Biology 25, no. 4 (2018): 380–391, 10.1016/j.chembiol.2018.01.005. 29396290 PMC6560181

[32] I. Helander and T. Mattila‐Sandholm , “Fluorometric Assessment of Gram‐Negative Bacterial Permeabilization,” Journal of Applied Microbiology 88, no. 2 (2000): 213–219, 10.1046/j.1365-2672.2000.00971.x. 10735988

[33] J. A. Karas , G. P. Carter , B. P. Howden , et al., “Structure–Activity Relationships of Daptomycin Lipopeptides,” Journal of Medicinal Chemistry 63, no. 22 (2020): 13266–13290, 10.1021/acs.jmedchem.0c00780. 32687352

[34] J. H. Jiang , M. S. Bhuiyan , H. H. Shen , et al., “Antibiotic Resistance and Host Immune Evasion in Staphylococcus aureus Mediated by A Metabolic Adaptation,” Proceedings of the National Academy of Sciences 116, no. 9 (2019): 3722–3727, 10.1073/pnas.1812066116. PMC6397524 30808758

[35] A. D. Berti , G. Sakoulas , V. Nizet , R. Tewhey , and W. E. Rose , “β‐Lactam Antibiotics Targeting PBP1 Selectively Enhance Daptomycin Activity against Methicillin‐Resistant Staphylococcus aureus,” Antimicrobial Agents and Chemotherapy 57, no. 10 (2013): 5005–5012, 10.1128/AAC.00594-13. 23896478 PMC3811465

[36] C. Liu , A. Bayer , S. E. Cosgrove , et al., “Clinical Practice Guidelines by the Infectious Diseases Society of America for the Treatment of Methicillin‐Resistant Staphylococcus Aureus Infections in Adults and Children,” Clinical infectious diseases 52, no. 3 (2011): e18–e55, 10.1093/cid/ciq146. 21208910

[37] F. Elías‐Wolff , M. Lindén , A. P. Lyubartsev , et al., “Curvature Sensing by Cardiolipin in Simulated Buckled Membranes,” Soft Matter 15, no. 4 (2019): 792–802, 10.1039/C8SM02133C. 30644502

[38] J. M. Alakoskela , M. J. Parry , and P. K. Kinnunen , “The Intermediate State of DMPG is Stabilized by Enhanced Positive Spontaneous Curvature,” Langmuir 26, no. 7 (2010): 4892–4900, 10.1021/la100411p. 20205407

[39] N. W. Schmidt and G. C. Wong , “Antimicrobial Peptides and Induced Membrane Curvature: Geometry, Coordination Chemistry, and Molecular Engineering,” Current Opinion in Solid State and Materials Science 17, no. 4 (2013): 151–163, 10.1016/j.cossms.2013.09.004. 24778573 PMC4000235

[40] I. R. Cooke and M. Deserno , “Coupling Between Lipid Shape and Membrane Curvature,” Biophysical Journal 91, no. 2 (2006): 487–495, 10.1529/biophysj.105.078683. 16807230 PMC1483084

[41] D. Koller and K. Lohner , “The Role of Spontaneous Lipid Curvature in the Interaction of Interfacially Active Peptides with Membranes,” Biochimica et Biophysica Acta (BBA)—Biomembranes 1838, no. 9 (2014): 2250–2259, 10.1016/j.bbamem.2014.05.013. 24853655

[42] Y. Ding , S. H. Chow , J. Chen , et al., “Targeted Delivery of LM22A‐4 by Cubosomes Protects Retinal Ganglion Cells in an Experimental Glaucoma Model,” Acta Biomaterialia 126 (2021): 433–444, 10.1016/j.actbio.2021.03.043. 33774200

[43] X. Lai , S. H. Chow , A. P. Le Brun , et al., “Polysaccharide‐Targeting Lipid Nanoparticles to Kill Gram‐Negative Bacteria,” Small 20 (2023): 2305052, 10.1002/smll.202305052. 37798622

[44] J. Chen , H. Zhou , J. Huang , R. Zhang , and X. Rao , “Virulence Alterations in Staphylococcus Aureus Upon Treatment with the Sub‐Inhibitory Concentrations of Antibiotics,” Journal of Advanced Research 31 (2021): 165–175, 10.1016/j.jare.2021.01.008. 34194840 PMC8240104

[45] R. Molotkovsky and P. Bashkirov , “Influence of Membrane Curvature on the Energy Barrier of Pore Formation,” Biochemistry (Moscow), Supplement Series A: Membrane and Cell Biology 18, no. 1 (2024): S1–S11, 10.1134/S1990747825700011.

[46] X. Lai , Y. Ding , C. M. Wu , et al., “Phytantriol‐Based Cubosome Formulation as an Antimicrobial Against Lipopolysaccharide‐Deficient Gram‐Negative Bacteria,” ACS Applied Materials & Interfaces 12, no. 40 (2020): 44485–44498, 10.1021/acsami.0c13309. 32942850

[47] M. L. Han , T. Velkov , Y. Zhu , et al., “Polymyxin‐Induced Lipid a Deacylation in Pseudomonas Aeruginosa Perturbs Polymyxin Penetration and Confers High‐Level Resistance,” ACS Chemical Biology 13, no. 1 (2018): 121–130, 10.1021/acschembio.7b00836. 29182311

[48] M. L. Han , H. H. Shen , K. A. Hansford , et al., “Investigating the Interaction of Octapeptin A3 with Model Bacterial Membranes,” ACS Infectious Diseases 3, no. 8 (2017): 606–619, 10.1021/acsinfecdis.7b00065. 28695731 PMC5955700

[49] H. H. Shen , V. Lake , A. P. Le Brun , et al., “Targeted Detection of Phosphatidylserine in Biomimetic Membranes and In Vitro Cell Systems Using Annexin V‐Containing Cubosomes,” Biomaterials 34, no. 33 (2013): 8361–8369, 10.1016/j.biomaterials.2013.07.042. 23899446

[50] X. Lai , L. Yu , X. Huang , et al., “Enhanced Nitric Oxide Delivery Through Self‐Assembling Nanoparticles for Eradicating Gram‐Negative Bacteria,” Advanced Healthcare Materials 13 (2024): 2403046, 10.1002/adhm.202403046. 39263842 PMC11670277

[51] E. J. Culp , N. Waglechner , W. Wang , et al., “Evolution‐Guided Discovery of Antibiotics that Inhibit Peptidoglycan Remodelling,” Nature 578, no. 7796 (2020): 582–587, 10.1038/s41586-020-1990-9. 32051588

[52] A. Khosravanizadeh , P. Sens , and F. Mohammad‐Rafiee , “Role of Particle Local Curvature in Cellular Wrapping,” Journal of the Royal Society Interface 19, no. 196 (2022): 20220462, 10.1098/rsif.2022.0462. 36321371 PMC9627444

[53] X. Gao , J. Dong , and X. Zhang , “The Effect of Nanoparticle Size on Endocytosis Dynamics Depends on Membrane–Nanoparticle Interaction,” Molecular Simulation 41, no. 7 (2015): 531–537, 10.1080/08927022.2014.896995.

[54] T. E. Hartnett , K. Ladewig , A. J. O'Connor , P. G. Hartley , and K. M. McLean , “Size and Phase Control of Cubic Lyotropic Liquid Crystal Nanoparticles,” The Journal of Physical Chemistry B 118, no. 26 (2014): 7430–7439, 10.1021/jp502898a. 24915497

[55] E. Rascol , J. M. Devoisselle , and J. Chopineau , “The Relevance of Membrane Models to Understand Nanoparticles–Cell Membrane Interactions,” Nanoscale 8, no. 9 (2016): 4780–4798, 10.1039/C5NR07954C. 26868717

[56] H. Heerklotz , “Membrane Stress and Permeabilization Induced by Asymmetric Incorporation of Compounds,” Biophysical Journal 81, no. 1 (2001): 184–195, 10.1016/S0006-3495(01)75690-1. 11423405 PMC1301502

[57] J. G. Hurdle , A. J. O'Neill , I. Chopra , et al., “Targeting Bacterial Membrane Function: An Underexploited Mechanism for Treating Persistent Infections,” Nature Reviews Microbiology 9, no. 1 (2011): 62–75, 10.1038/nrmicro2474. 21164535 PMC3496266

[58] P. Astolfi , E. Giorgini , F. C. Adamo , et al., “Effects of a Cationic Surfactant Incorporation in Phytantriol Bulk Cubic Phases and Dispersions Loaded with the Anticancer Drug 5‐Fluorouracil,” Journal of Molecular Liquids 286 (2019): 110954, 10.1016/j.molliq.2019.110954.

[59] A. P. Le Brun , T. Y. Huang , S. Pullen , et al., “Spatz: The Time‐of‐Flight Neutron Reflectometer with Vertical Sample Geometry at the OPAL Research Reactor,” Journal of Applied Crystallography 56, no. 1 (2023): 18–25, 10.1107/S160057672201086X. 36777140 PMC9901927

[60] M. James , A. Nelson , S. A. Holt , et al., “The Multipurpose Time‐of‐Flight Neutron Reflectometer “platypus” at Australia's Opal Reactor,” Nuclear Instruments and Methods in Physics Research Section A: Accelerators, Spectrometers, Detectors and Associated Equipment 632, no. 1 (2011): 112–123, 10.1016/j.nima.2010.12.075.

[61] A. R. J. Nelson and S. W. Prescott , “refnx: Neutron and X‐Ray Reflectometry Analysis in Python,” Journal of Applied Crystallography 52, no. Pt 1 (2019): 193–200, 10.1107/S1600576718017296. 30800030 PMC6362611

[62] S. A. Holt , T. E. Oliver , and A. R. Nelson , “Using refnx to Model Neutron Reflectometry Data from Phospholipid Bilayers,” in Membrane Lipids: Methods and Protocols (Springer, 2022), 179–197, 10.1007/978-1-0716-1843-1_15. 34854045

