# Supplementary material for: Enhanced Antibacterial Properties of Lyotropic Liquid Crystalline Nanoparticles via Curvature Modulation
Source: Adv Sci (Weinh). 2026 Jul 9:e76516. Online ahead of print. doi: 10.1002/advs.76516 (PMC13348654; doi:10.1002/advs.76516)
Supplement: Supplementary file 1 — Supporting File: advs76516‐sup‐0001‐SuppMat.docx. [file ADVS-9999-e76516-s001.docx]

Supplementary Materials for

**Enhanced Antibacterial Properties of Lyotropic Liquid Crystalline Nanoparticles** **via Curvature Modulation**

Xiangfeng Lai, Shuhong Wang, Chenguang Ding, Xenia Kostoulias, Anton P. Le Brun, Hsien-Yi Hsu, Jhih-Hang Jiang, Yajun Wang, Richard A. Strugnell, Anton Y. Peleg*, and Hsin-Hui Shen*

Supplementary Materials for this manuscript include the following:

Supplementary Figures. S1 to S17

Supplementary Tables S1 to S10


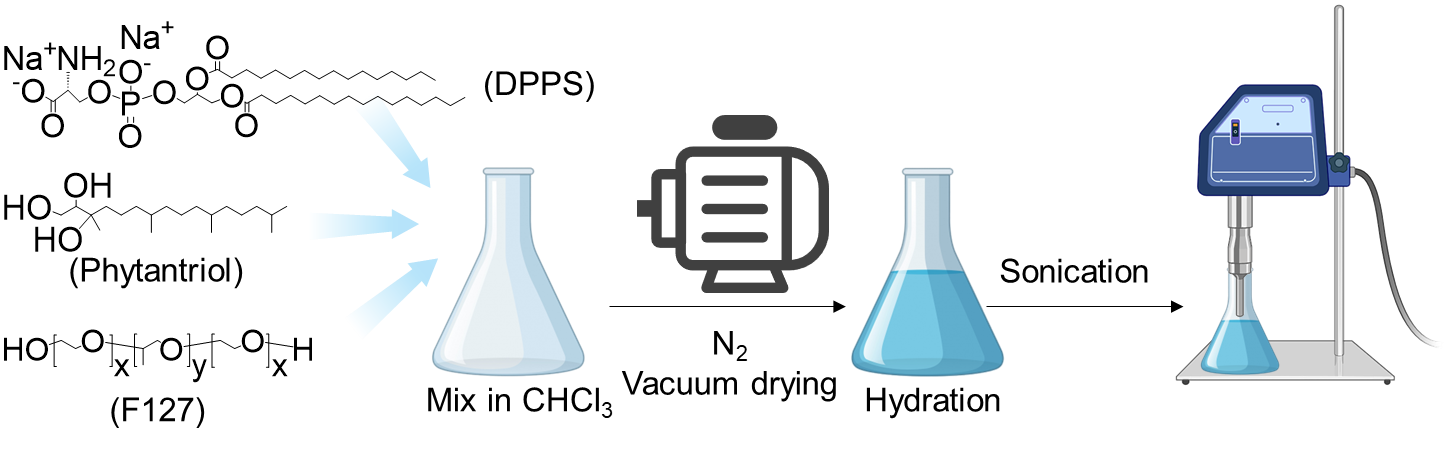


**Supplementary Figure S1. Formulation process.** LCNPs were formulated by a top-down process involving lipid mixing, N_2_/vacuum drying, water hydration and tip sonication.


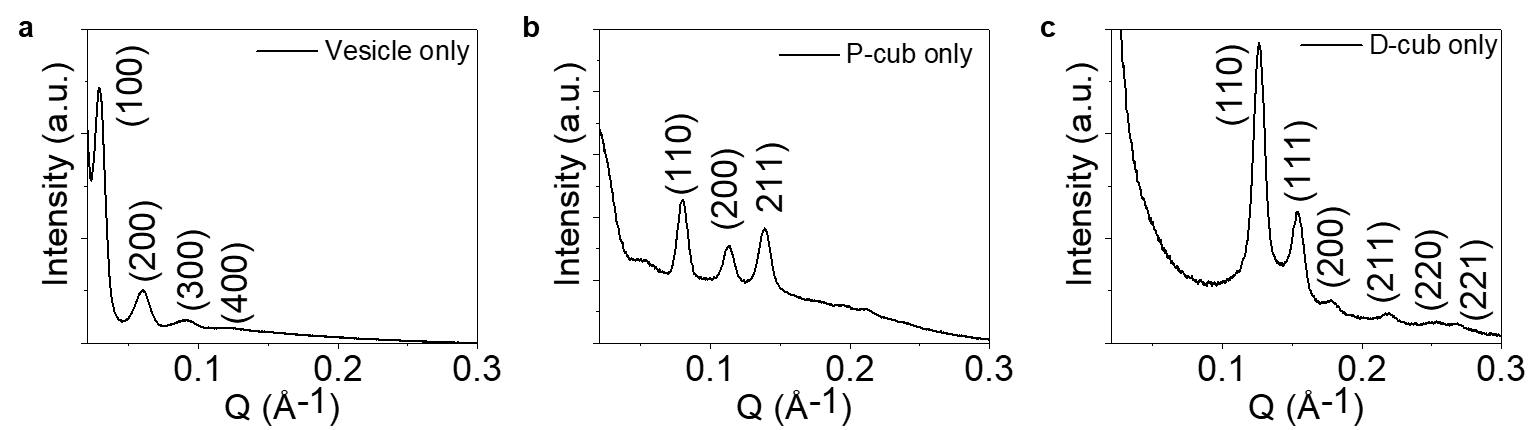


**Supplementary Figure S2.** SAXS profiles for (**a**) vesicles, (**b**) P-cubosomes and (**c**) D-cubosomes in the same final cell culture medium. a. u., arbitrary unit.


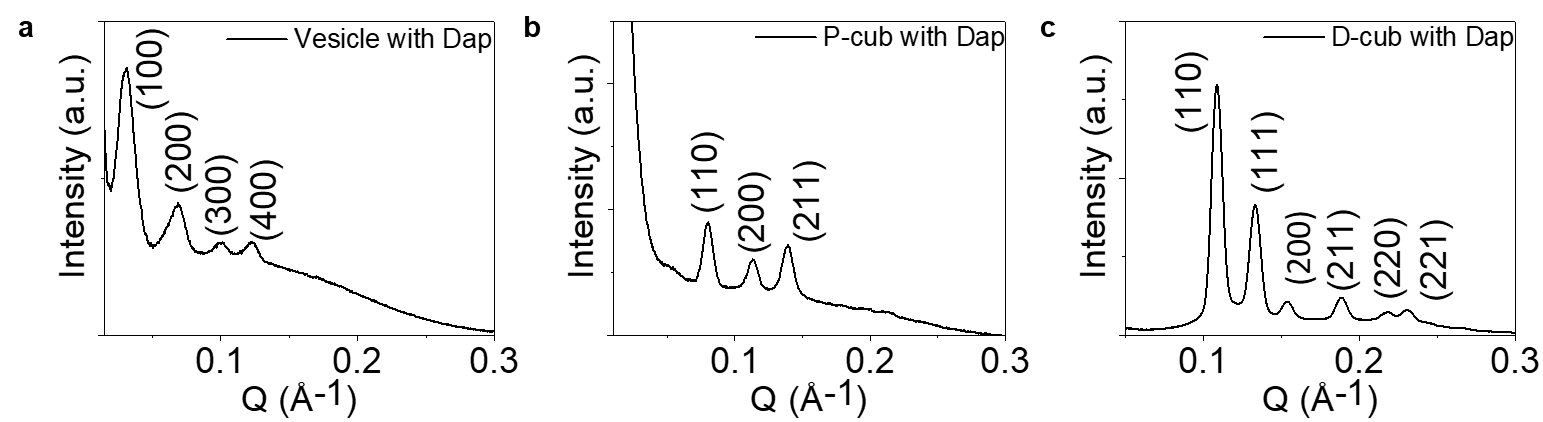


**Supplementary Figure S3.** SAXS profiles for (**a**) vesicles, (**b**) P-cubosomes and (**c**) D-cubosomes co-incubate with 0.25 μg/mL daptomycin (Dap) at 37 °C for 24 h in the same final cell culture medium. Bragg series/indexing are unchanged. a. u., arbitrary unit.

**
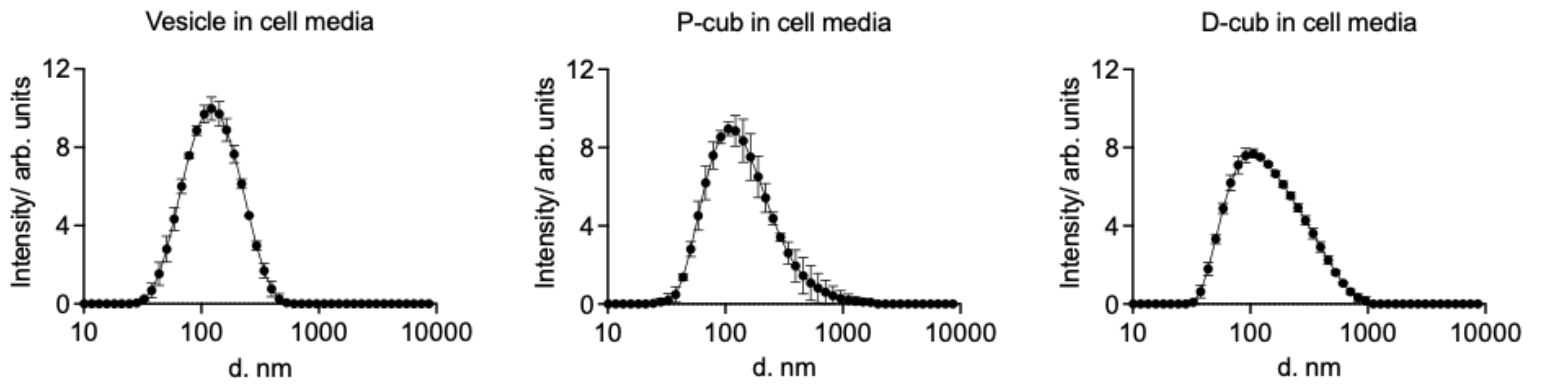
**

**Supplementary Figure S4.** The size distribution of LCNPs in cell culture media. n = 3.

**
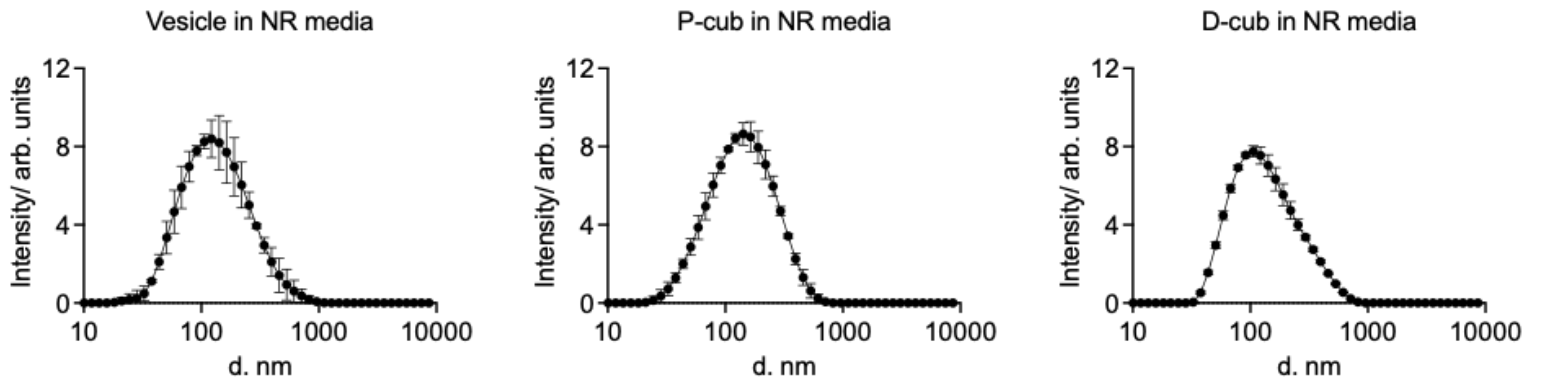
**

**Supplementary Figure S5.** The size distribution of LCNPs in CaCl₂/NaCl/ HEPES buffer. n = 3.

**
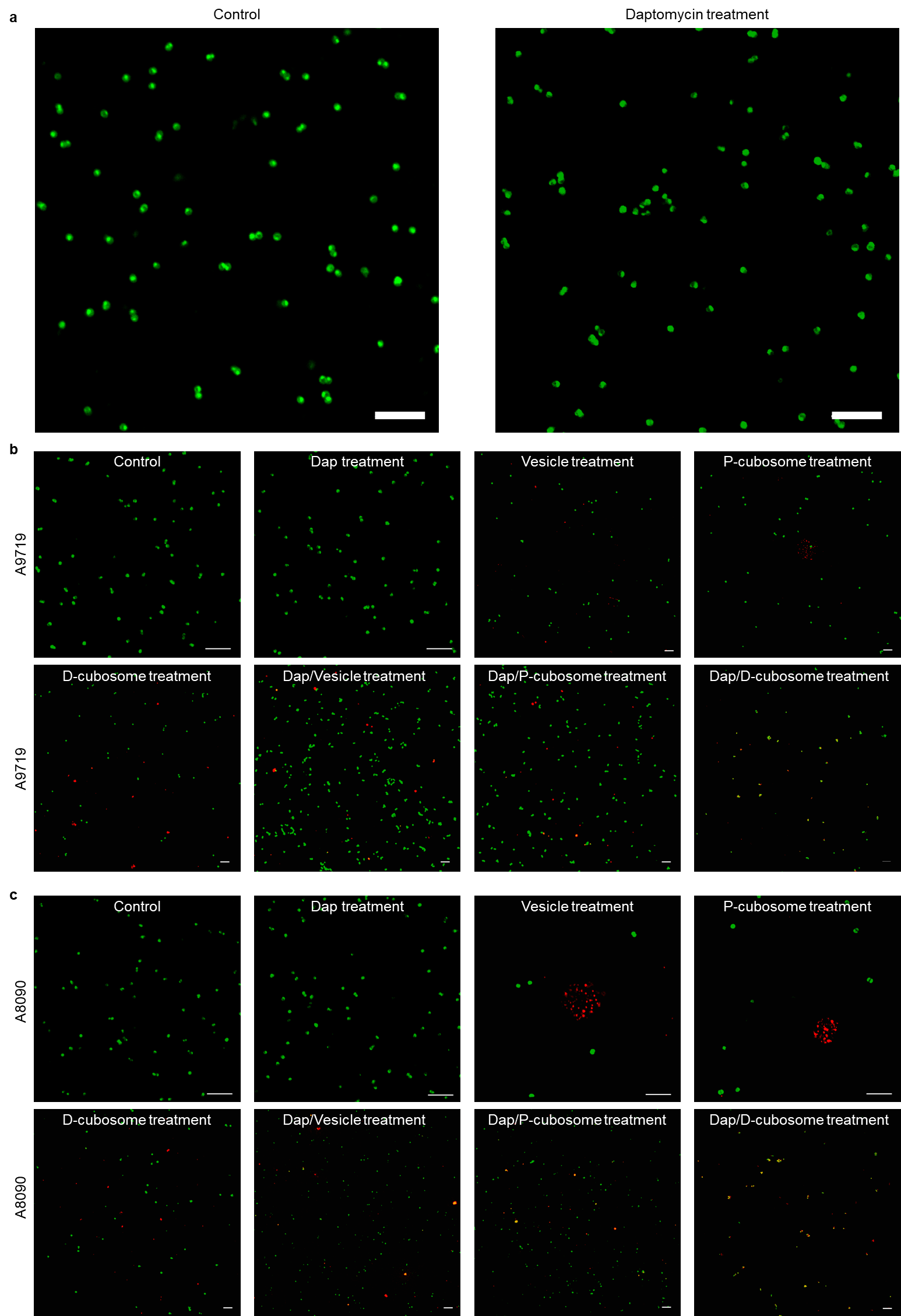
**

**
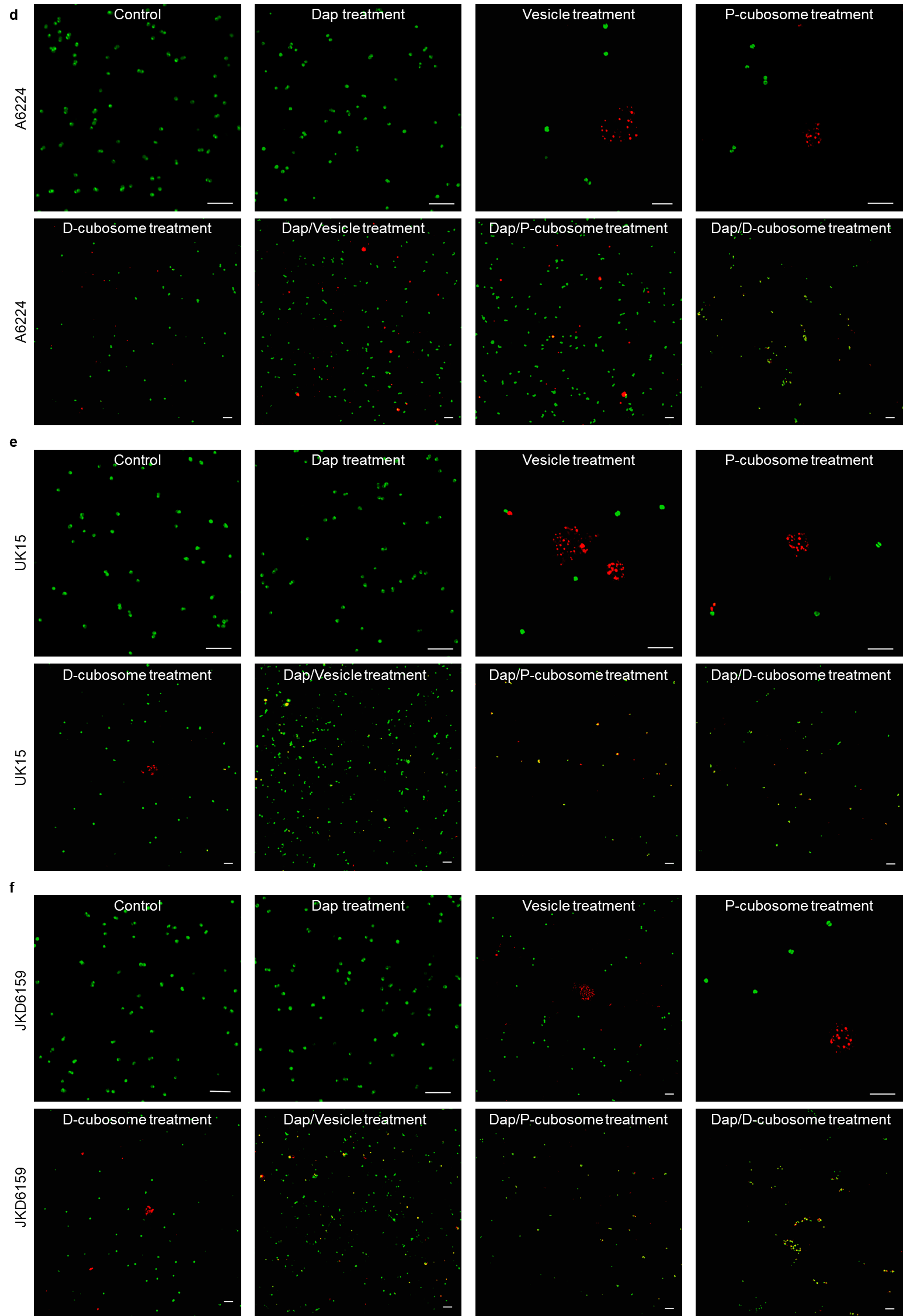
**

**Supplementary Figure S6. LCNP – membrane interaction study.** Fluorescence microscopy images of six MRSA strains (control) under different treatments. **a** A8819. **b** A9719. **c** A8090. **d** A6224. **e** UK15. **f** JKD6159. Scale bar: 5 µm. All images are representative of three independent experiments.

**
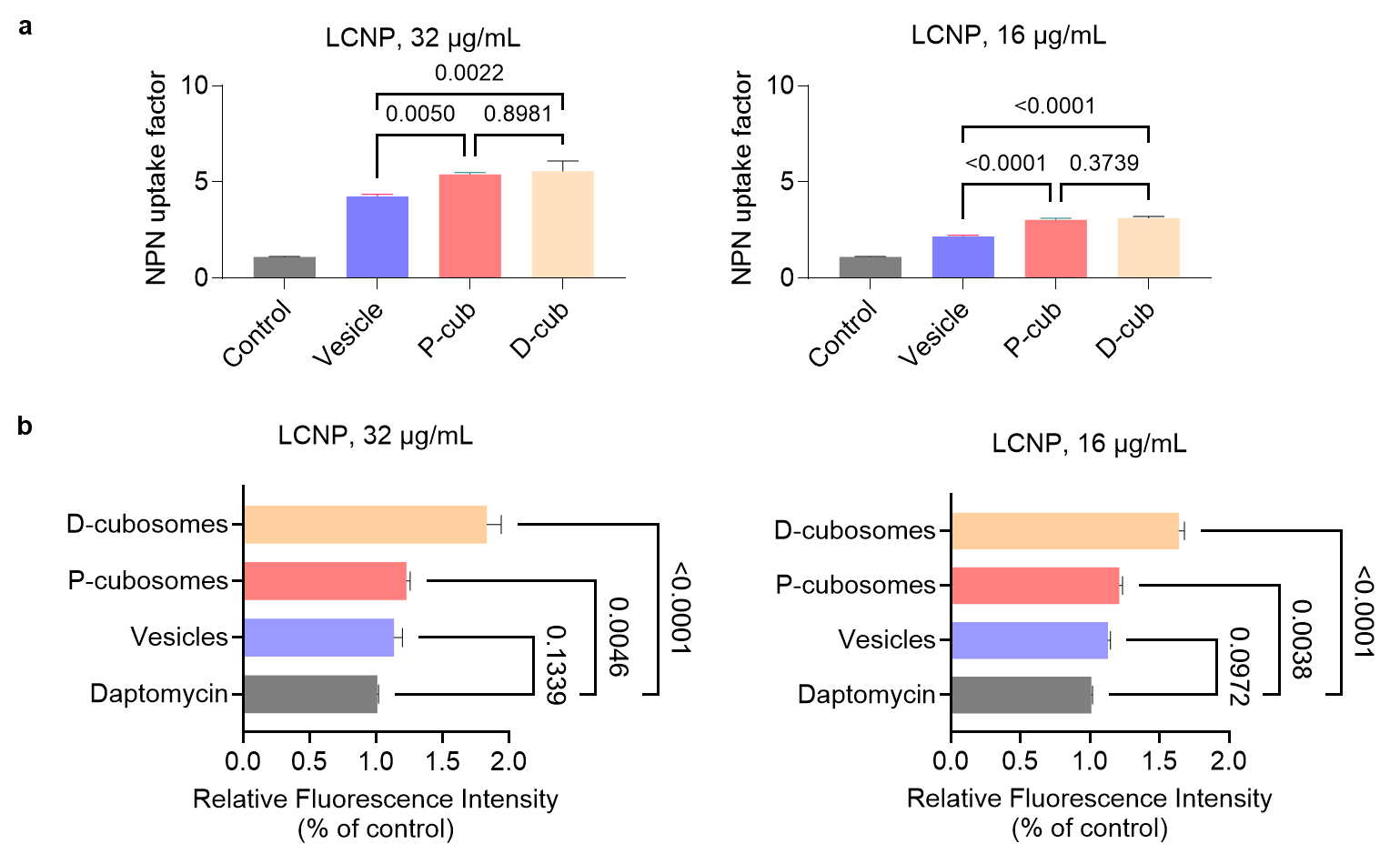
**

**Supplementary Figure S7. Interaction of LCNPs alone with MRSA *A8819*** **membrane. a** MRSA *A8819* membrane permeabilization as measured with 1-N-phenylnaphthylamine (NPN) fluorescence emission (Ex/Em: 340 nm/ 405 nm). Data points represent mean ± SD (n = 3). **b** The relative fluorescence intensity generated at 24h under different treatments in 2',7'-dichlorofluorescin diacetate-stained MRSA A8819. Daptomycin (Dap, 0.25 ug/mL), LCNPs: 16-32 ug/mL. Error bars represent the mean ± SD (n = 3). Statistical significance was determined using one-way ANOVA.

**
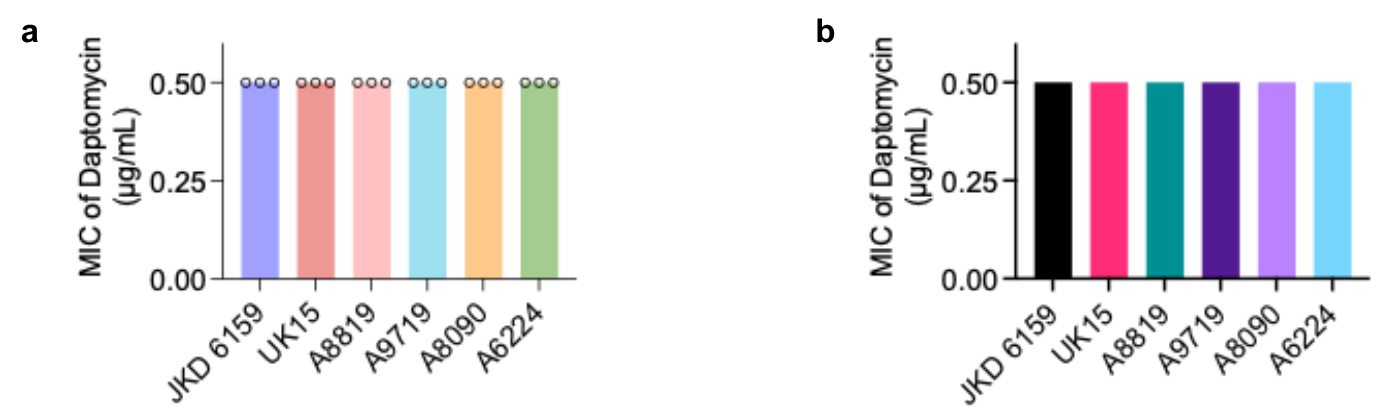
**

**Supplementary Figure S8. Antimicrobial activity assay. a** The minimum inhibitory concentration (MIC) of daptomycin alone against six MRSA strains (n=3). **b** Daptomycin MIC as a function of ionic strength in 10 mM HEPES (pH 7.4) containing NaCl at 0, 15 (mimics 0.1X PBS), 75, or 150 (mimics 1X PBS/neutron buffer) mM (final concentrations after inoculation). The control experiment revealed that 0.1X PBS 1X PBS did not affect the bacterial growth.


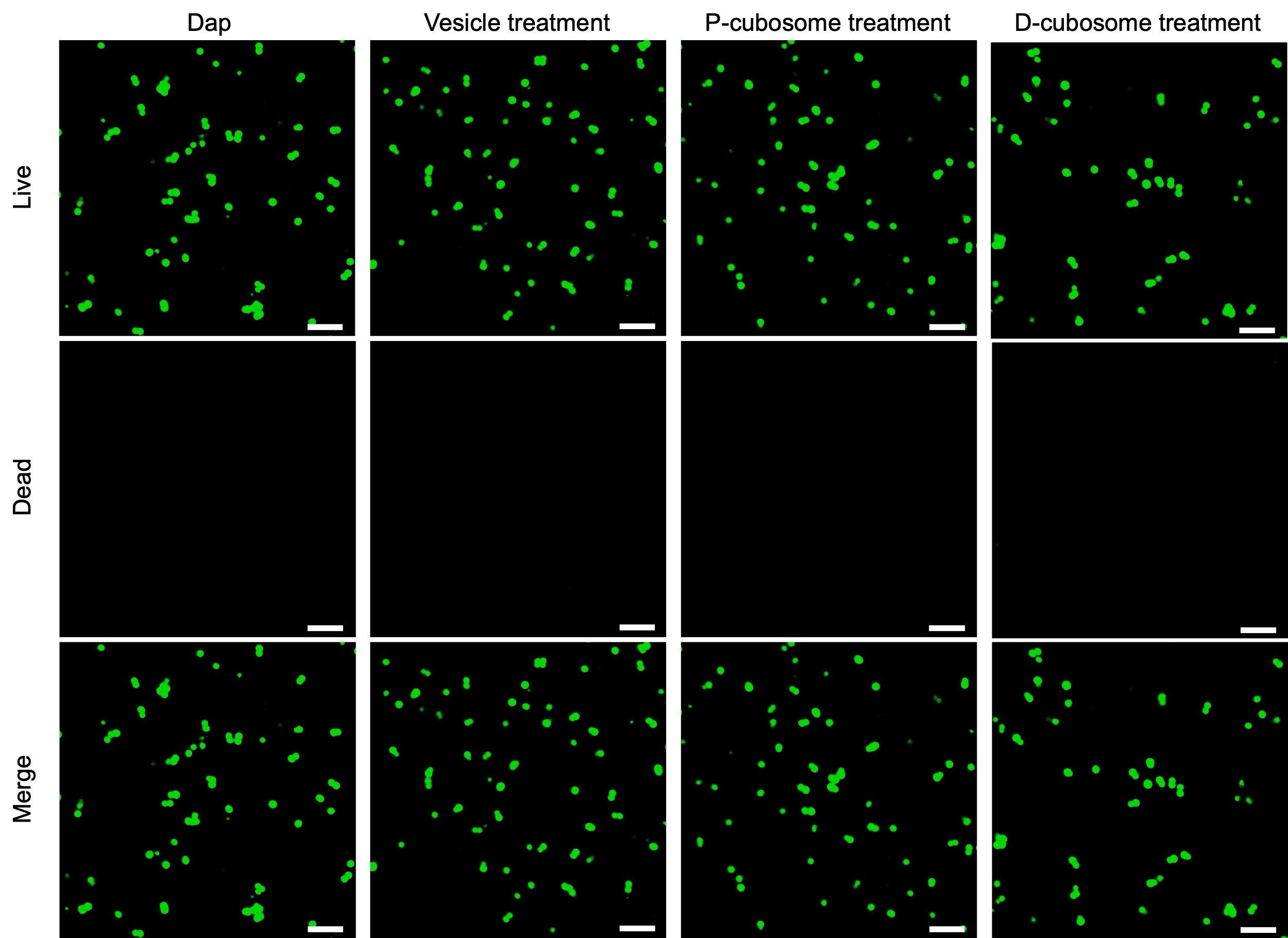


**Supplementary Figure S9.** **Representative fluorescent microscopy images.** Live/dead staining of MRSA A8819 treated with Daptomycin (Dap) and LCNP monotherapies. LCNP-only treatments show minimal propidium iodide uptake and no abnormal fluorescence signals, confirming negligible interference of LCNPs with SYTO 9/PI staining. Scale bars, 5 μm.

**Supplementary Figure S10.** Analysis of relative viability of *S. aureus* suspensions in a fluorescence microplate reader. The integrated intensities of the green (530 ± 30 nm) and red (640 ± 30 nm) emission of suspensions excited at 480 ± 30 nm were acquired, and the green/red fluorescence ratios were calculated for each proportion of live/dead *S. aureus*. Each point represents the mean of three measurements. The line is a least-squares fit of the relationship between % live bacteria (x) and green/red ratio (y).


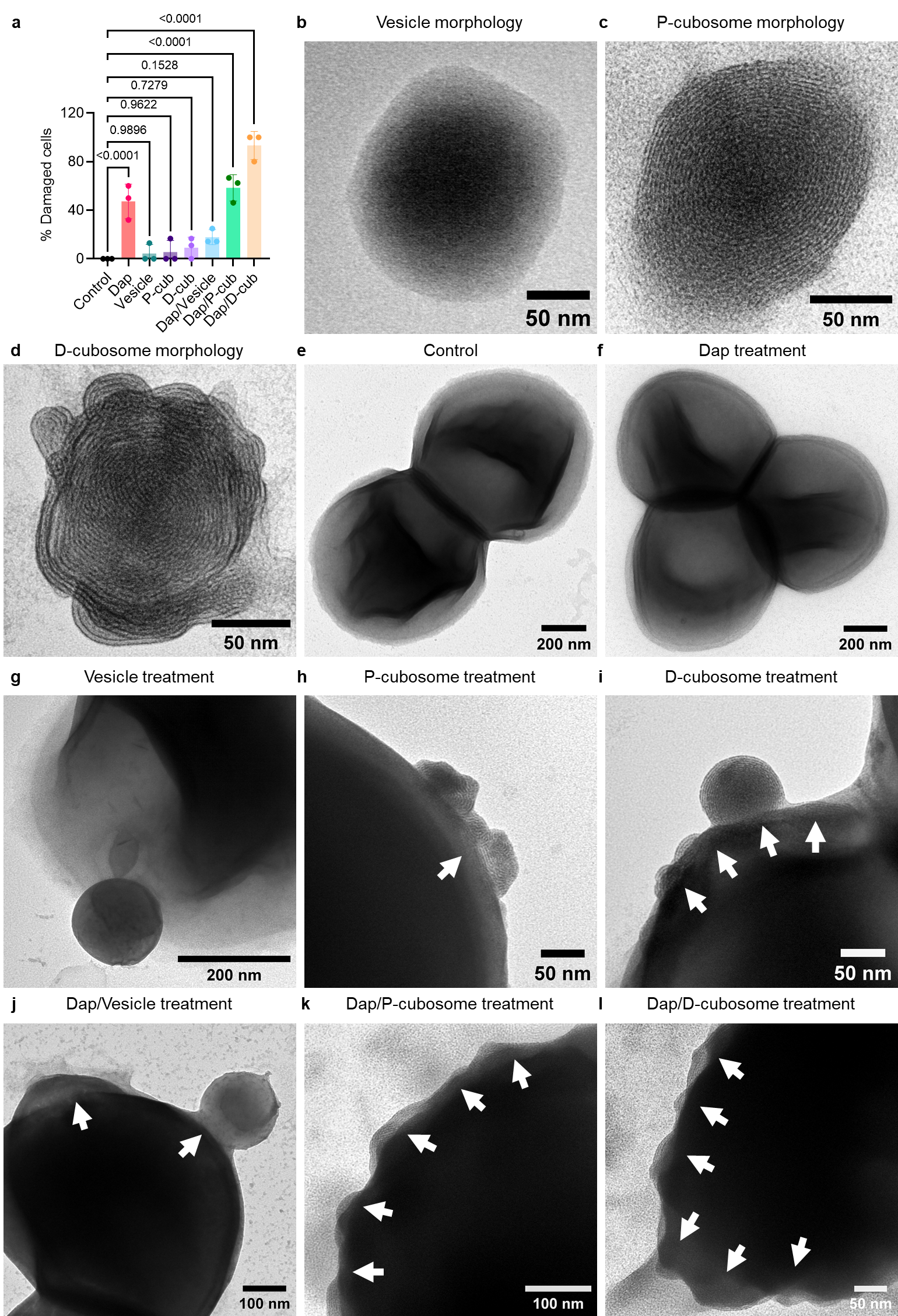


**Supplementary Figure S11.** Electron Microscopy. **a** Semi-quantitative analysis of SEM images showing the percentage of damaged MRSA A8819 cells under different treatments. Cells were classified as damaged based on visible membrane deformation, blebbing, protrusions, or rupture. Data represent mean ± SD from three independent fields of view per condition. Statistical significance was determined using one-way ANOVA. Conventional TEM images of **b** Vesicles, **c** P-cubosomes, **d** D-cubosomes, **e** MRSA A8819 control and **f** MRSA A8819 treated with daptomycin (Dap). The enlarged TEM images for monotherapies of **g** vesicles, **h** P-cubosomes and **i** D-cubosomes. The enlarged TEM images for polytherapy of daptomycin with **j** vesicles, **k** P-cubosomes and **l** D-cubosomes. Regions of interest are indicated by white arrows. All images are representative of three independent experiments.

**Supplementary Figure S12.** *In vitro* cell viability of HEK 293T cells incubated in the presence of daptomycin (Dap), daptomycin, LCNPs and Dap (0.25 μg/mL) in combination with LCNPs. All Data are expressed as the mean ± SD. All experiments were performed in triplicate (n=3).


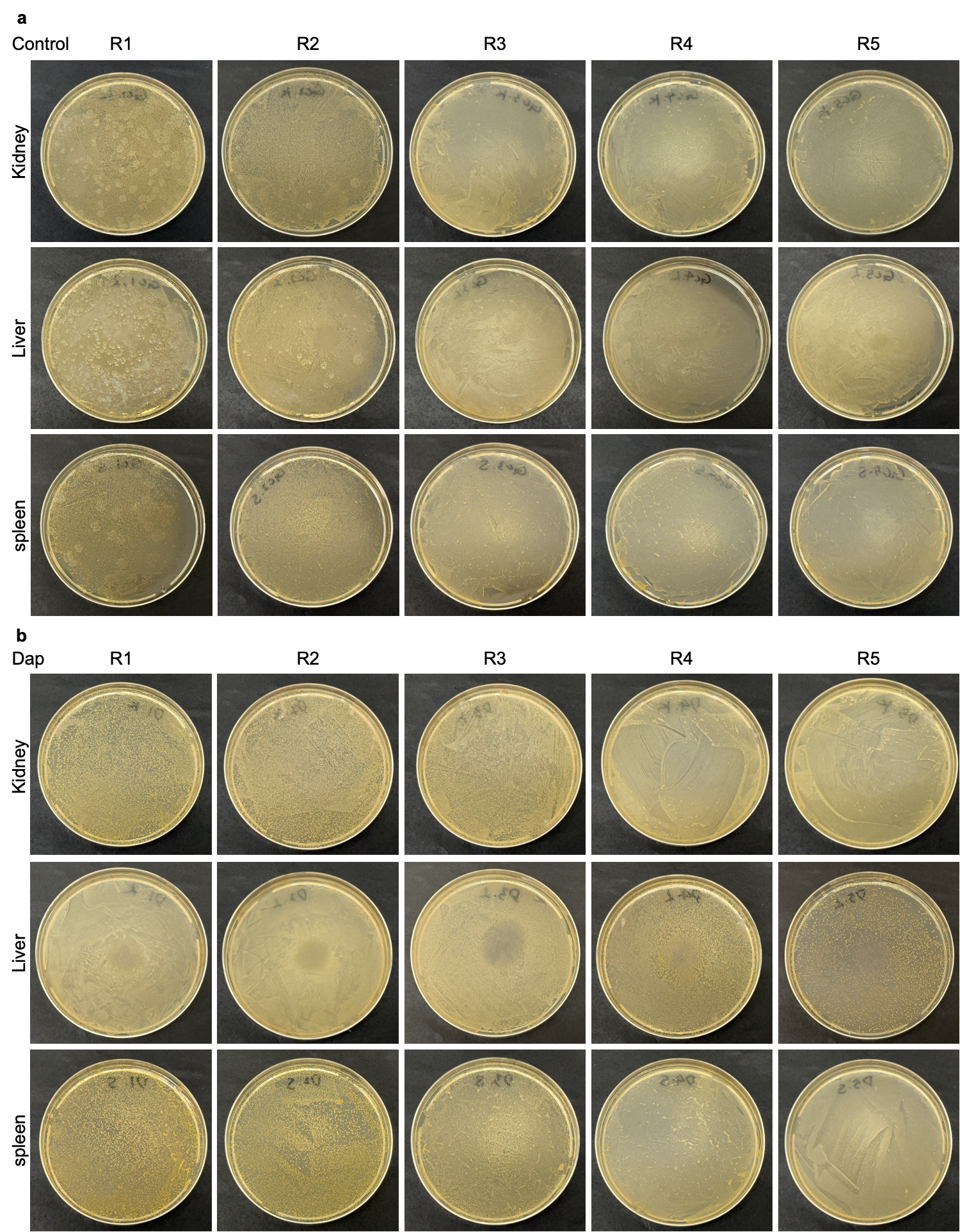


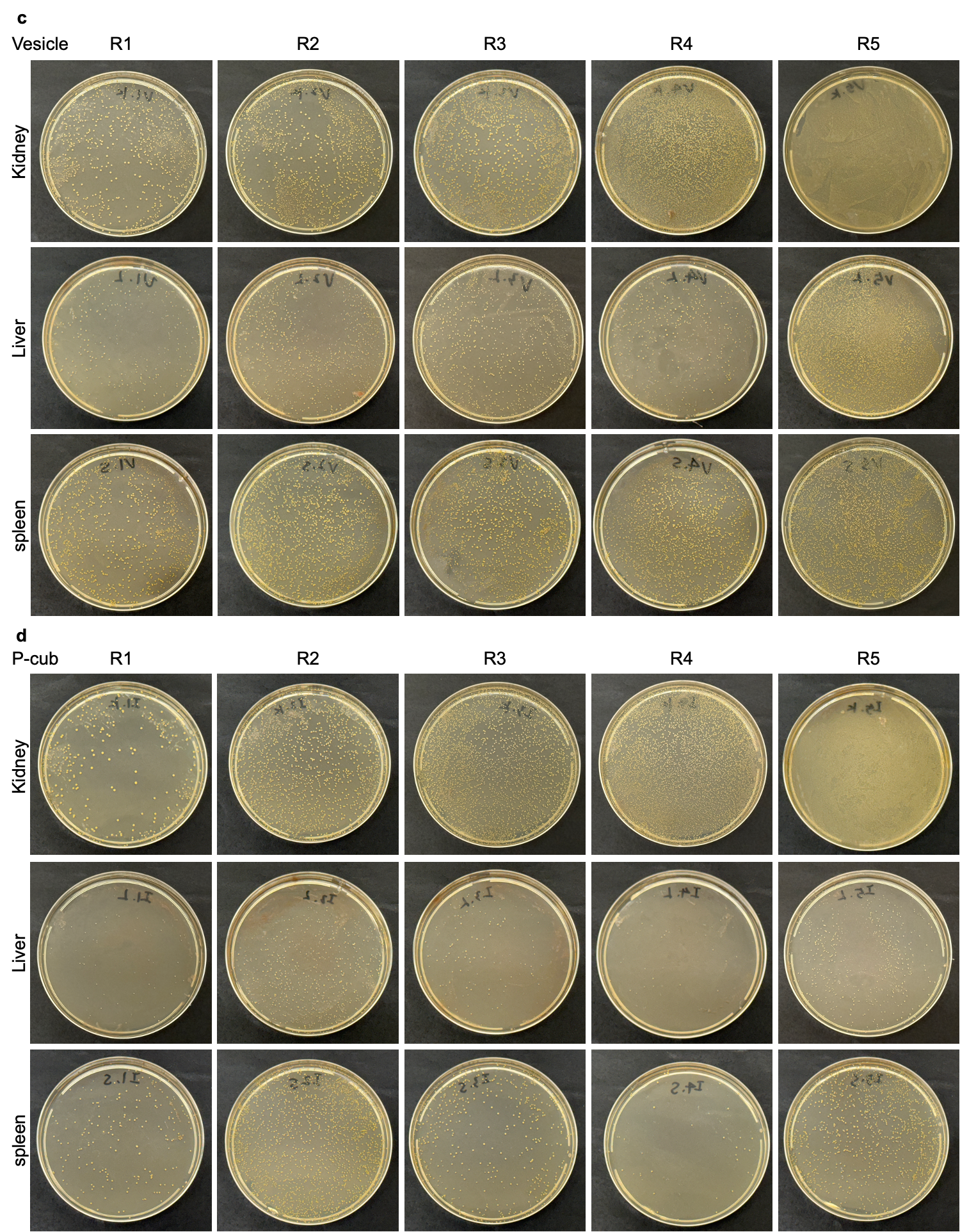


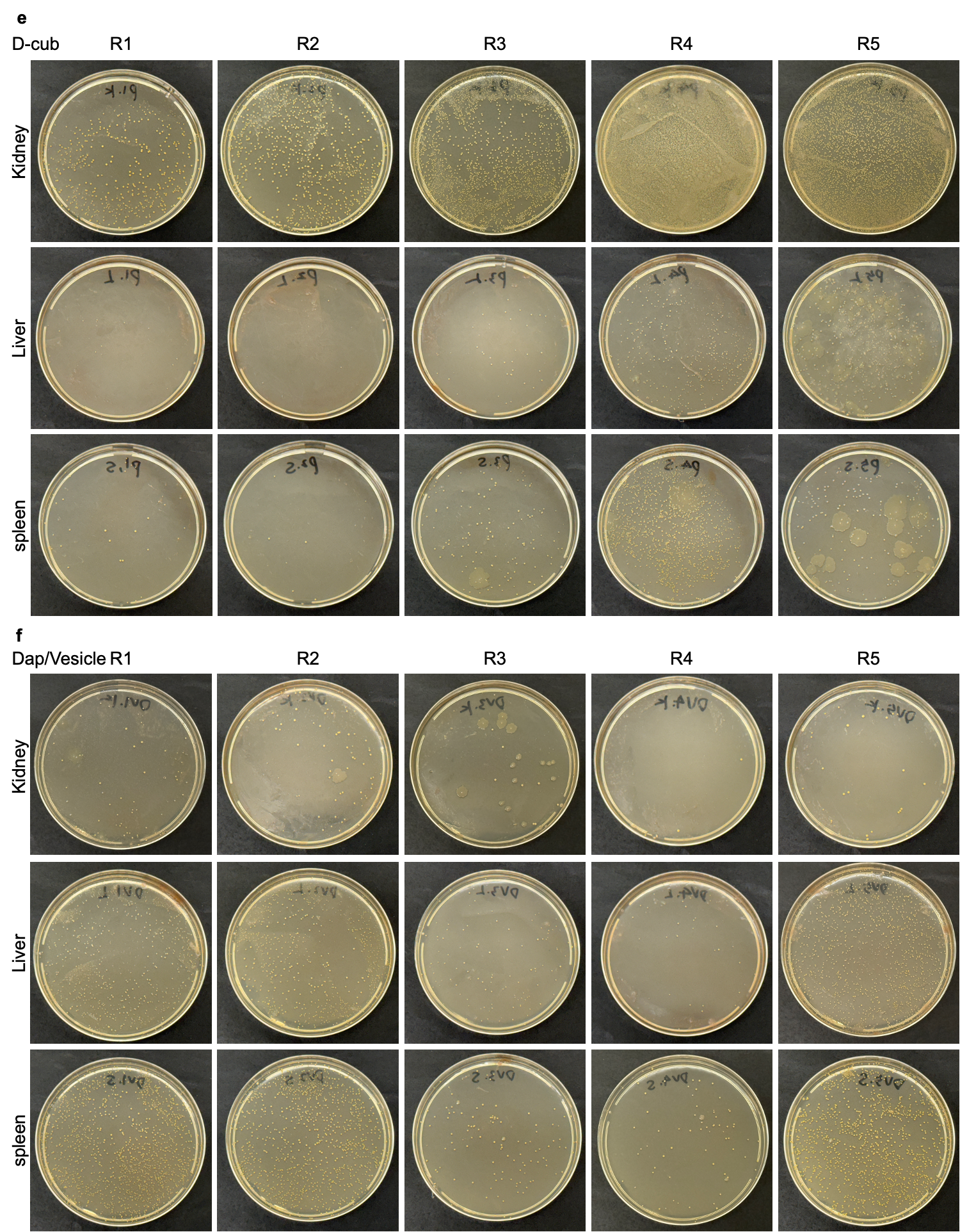


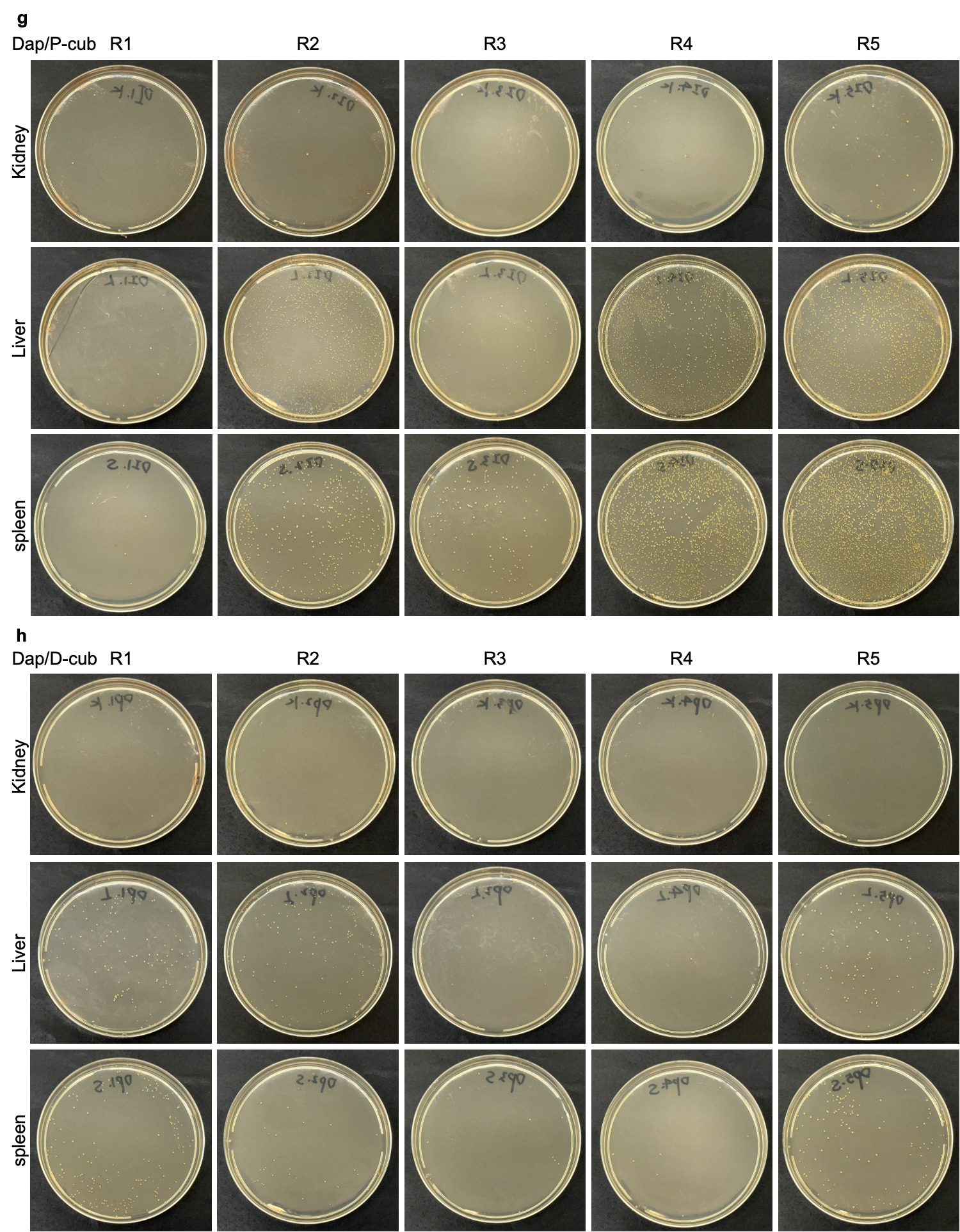


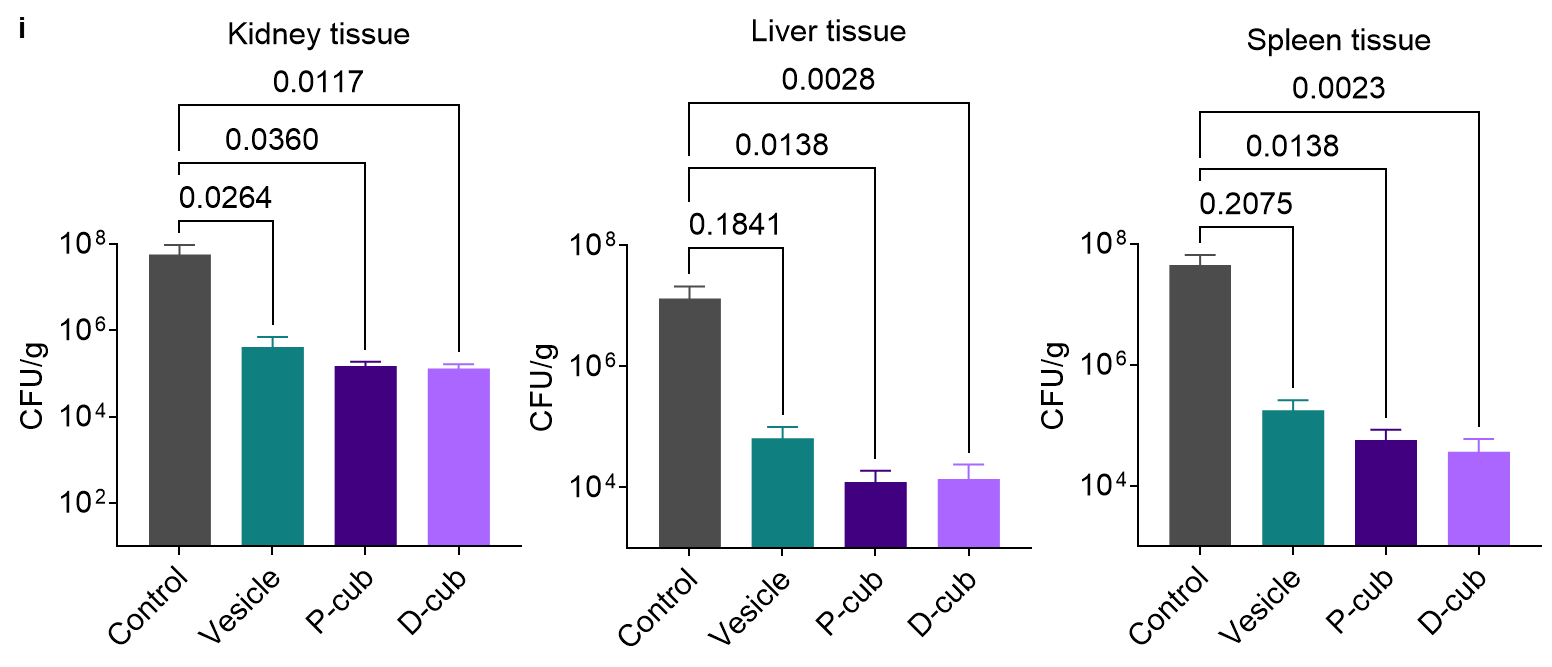


**Supplementary Figure S13. *In vivo* behaviours of bacteria in mice.** Homogenates of organs (kidney, liver and spleen) of mice after tail injection with (**a**) saline, (**b**) daptomycin (dap), (**c**) vesicles, (**d**) P-cubosomes (P-cub), (**e**) D-cubosomes (D-cub), (**f**) dap/vesicles, (**g**) dap/P-cub and (**h**) dap/D-cub for 24 h cultured on the solid MHB agar (n = 5). **i** Quantified colony forming unit (CFU) of *S. aureus* in excised organ tissues from treatment endpoints was performed (n = 5). Data are presented as means ± sem. Statistical significance was determined using one-way ANOVA.

**
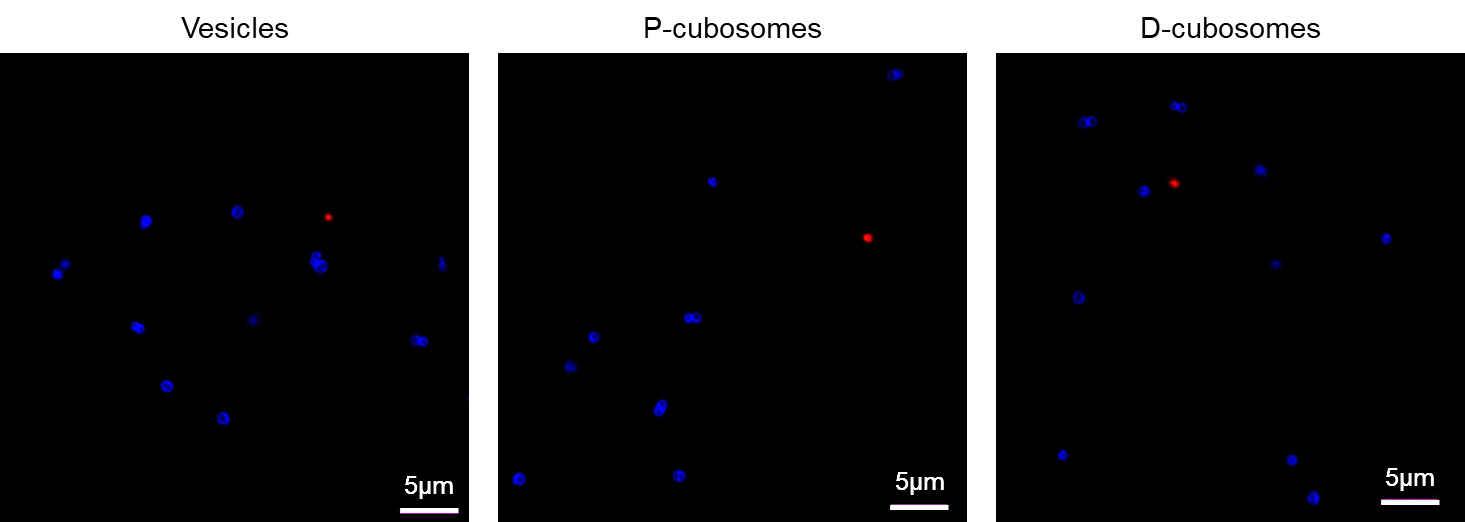
**

**Supplementary Figure S14. Interaction of LCNPs with peptidoglycan layer of MRSA A8819.** The peptidoglycan layer was stained with fluorescent D-amino acid probe - 7-hydroxycoumarin carbonyl amino-D-alanine (HADA, blue), and LCNPs with Rhodamine B (red) in all images.

**
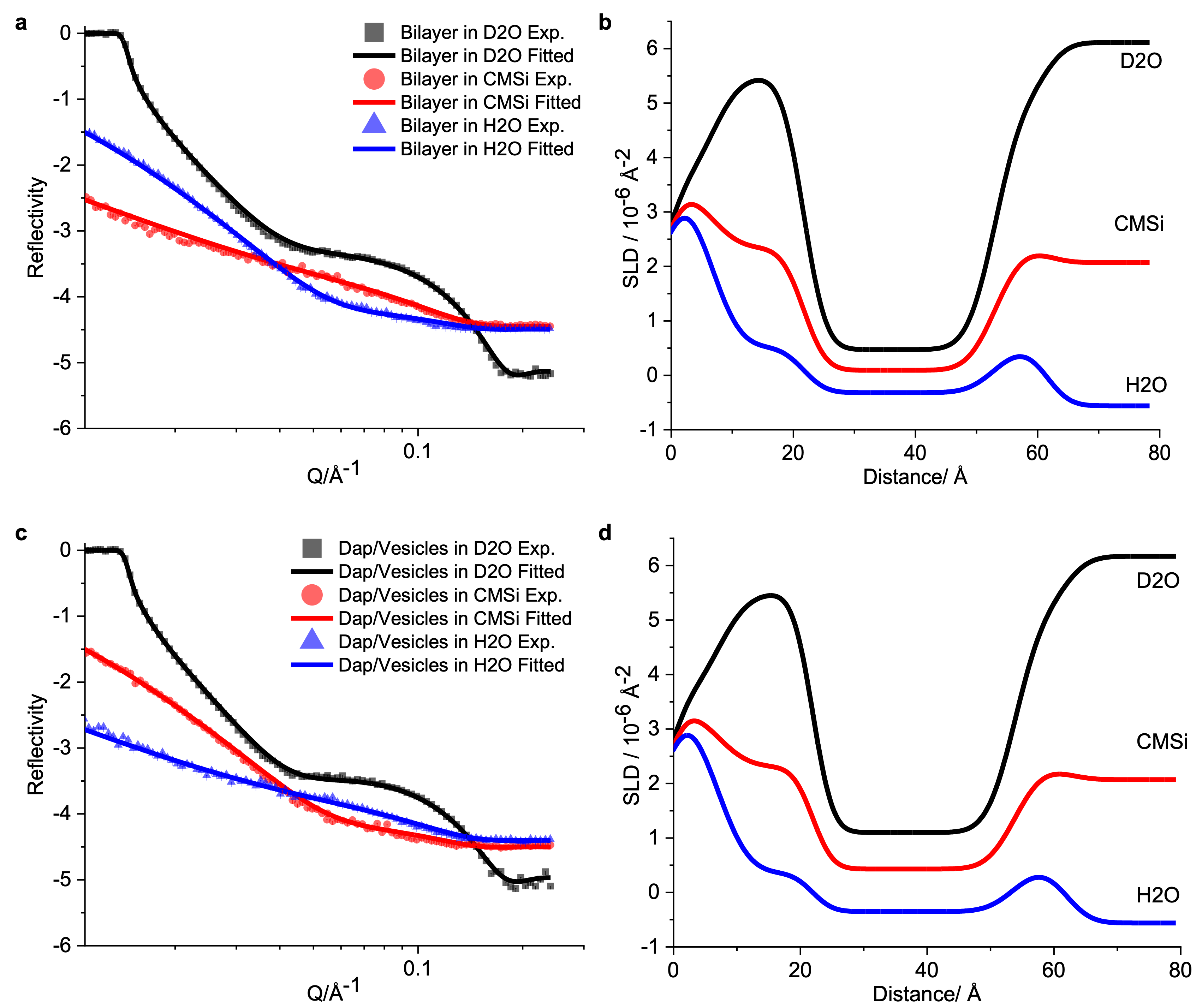
**

**Supplementary Figure S15. Neutron reflectometry profiles for model Gram-positive bacterial membrane treated with daptomycin (Dap)/Vesicles. a** Experimental (symbols) and fitted (lines) profiles of the model Gram-positive bacterial membrane bilayer. **b** Corresponding scattering length density (SLD) profiles of (**a**). **c** Reflectivity profiles after introducing daptomycin/Vesicles (2/ 64 µg/mL). **d** Corresponding scattering length density (SLD) profiles of (**c**). Error bars for the experimental data points are one standard deviation of the uncertainty in the reflectivity[1]. All data are expressed as median ± SD. (indicated by error bars), based on values obtained from three isotopic contrasts, *i.e.*, D_2_O, CMSi (contrast-matched silicon) and H_2_O.

**
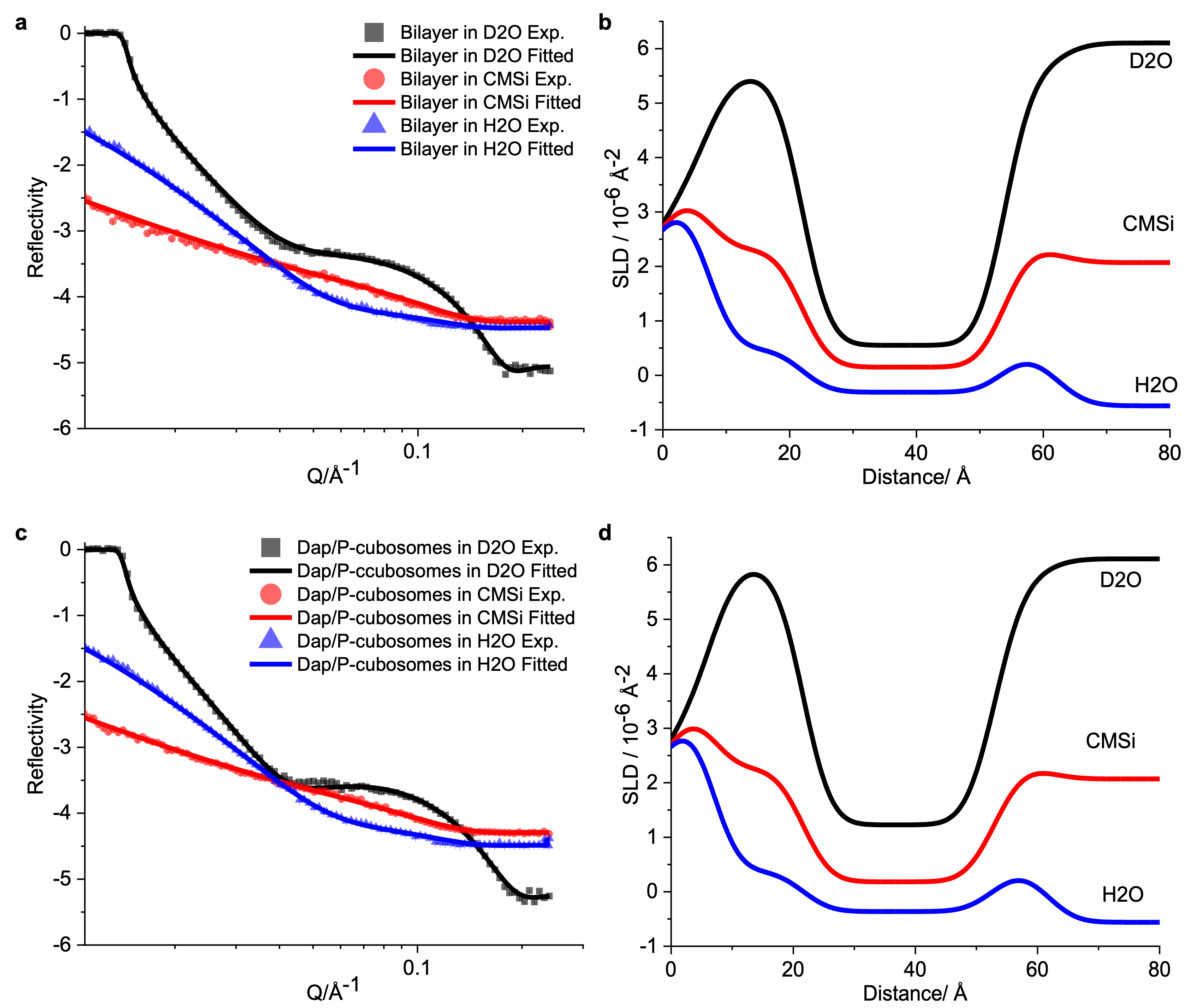
**

**Supplementary Figure S16. Neutron reflectometry profiles for model Gram-positive bacterial membrane treated with daptomycin (Dap)/P-cubosomes. a** Experimental (symbols) and fitted (lines) profiles of the model Gram-positive bacterial membrane bilayer. **b** Corresponding scattering length density (SLD) profiles of (**a**). **c** Reflectivity profiles after introducing daptomycin/P-cubosomes (2/ 64 µg/mL). **d** Corresponding scattering length density (SLD) profiles of (**c**). Error bars for the experimental data points are one standard deviation of the uncertainty in the reflectivity. All data are expressed as median ± SD. (indicated by error bars), based on values obtained from three isotopic contrasts, *i.e.*, D_2_O, CMSi (contrast-matched silicon) and H_2_O.

**
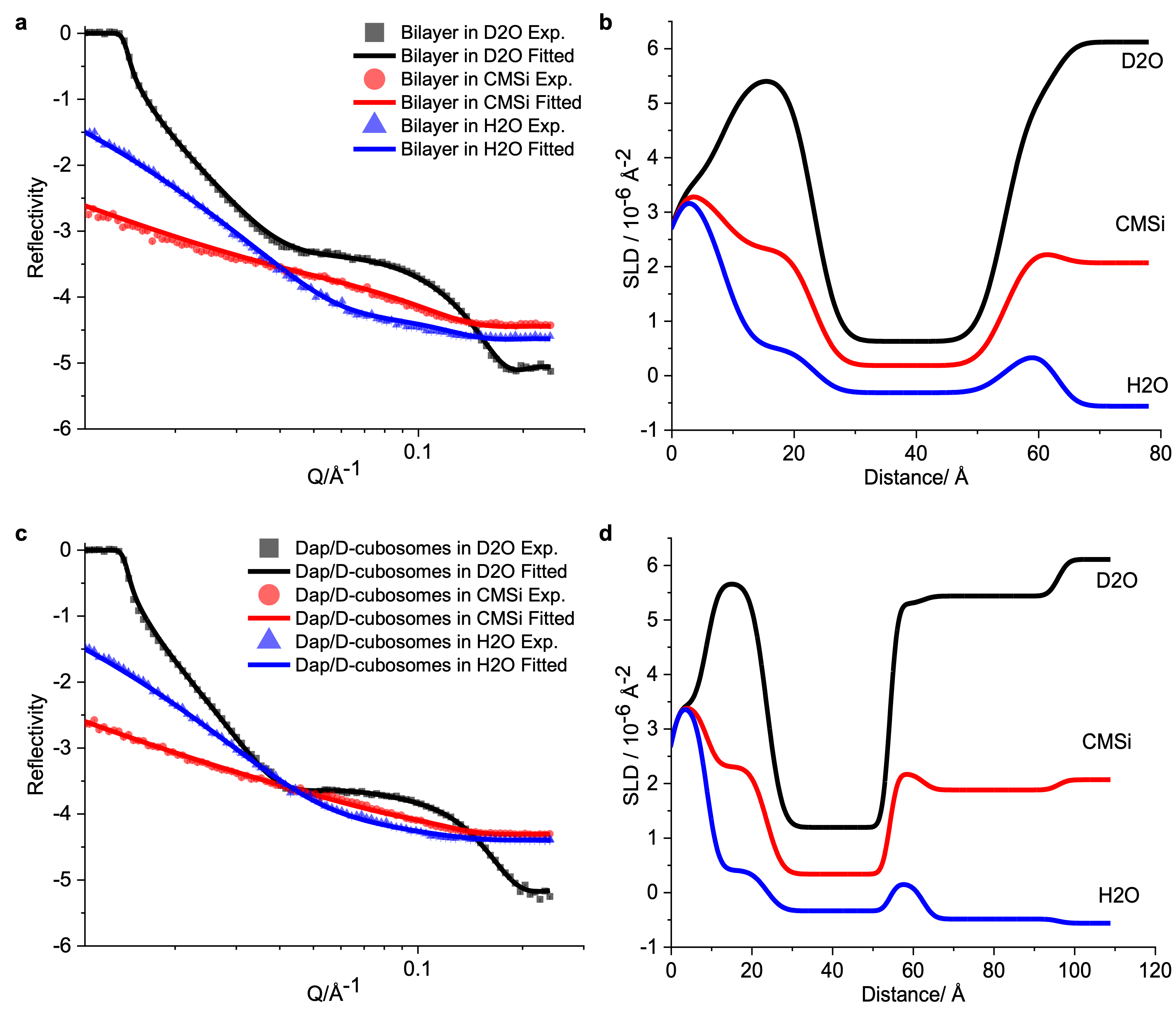
**

**Supplementary Figure S17. Neutron reflectometry profiles for model Gram-positive bacterial membrane treated with daptomycin (Dap)/D-cubosomes. a** Experimental (symbols) and fitted (lines) profiles of the model Gram-positive bacterial membrane bilayer. **b** Corresponding scattering length density (SLD) profiles of (**a**). **c** Reflectivity profiles after introducing daptomycin/D-cubosomes (2/ 64 µg/mL). **d** Corresponding scattering length density (SLD) profiles of (**c**). Error bars for the experimental data points are one standard deviation of the uncertainty in the reflectivity. All data are expressed as median ± SD. (indicated by error bars), based on values obtained from three isotopic contrasts, *i.e.*, D_2_O, CMSi (contrast-matched silicon) and H_2_O.

**Supplementary Table S1. DLS of LCNP in media used for neutron reflectometry experiment.**

| Nanoparticles | Z-average size (d, nm) | PDI | Zeta Potential (mV) |
| --- | --- | --- | --- |
| Vesicles | 113.7 ± 4.1 | 0.29 ± 0.01 | -4.1 ± 1.1 |
| P-cubosomes | 127.8 ± 4.1 | 0.23 ± 0.02 | -2.6 ± 1.5 |
| D-cubosomes | 134.4 ± 3.8 | 0.28 ± 0.01 | -2.9 ± 0.1 |
| For the comparative measurements, all LCNPs were first transferred into the same buffer (5 mM CaCl₂, 150 mM NaCl and 10 mM HEPES, pH 7.4) before analysis at 37 °C. n =4. | | | |

**Supplementary Table S2. DLS of combination of daptomycin with LCNP in assay media.**

| Nanoparticles | Z-average size (d, nm) | PDI | Zeta Potential (mV) |
| --- | --- | --- | --- |
| Vesicles | 109.7 ± 5.6 | 0.24 ± 0.04 | -4.1 ± 0.4 |
| P-cubosomes | 124.8 ± 3.2 | 0.20 ± 0.02 | -2.5 ± 0.2 |
| D-cubosomes | 132.9 ± 3.8 | 0.21 ± 0.03 | -1.8 ± 0.3 |

**Supplementary Table S3. Small angle X-ray scattering (SAXS).** Phase properties of 15 wt% DPPS LCNP dispersed in 0.1 X PBS and 1 X PBS buffer.

| Nanoparticles | 15 wt% DPPS | 15 wt% in 0.1X PBS | 15 wt% in 1X PBS |
| --- | --- | --- | --- |
| Space group | L*_α_* | Q_Ⅱ_^P^ (Im3m) | Q_Ⅱ_^D^ (Pn3m) |
| Unit cell parameter (*a*, nm) | 24.2 ± 0.1 | 12.7 ± 0.1 | 8.0 ± 0.1 |
| Surface-averaged Gaussian curvature (*K*, nm^-2^) | 0[2] | -0.07 ± 0.01 | -0.10 ± 0.01 |
| The unit cell parameter '*a*' was calculated using the formula: $a=d_{hkl}\sqrt{(h^{2}+k^{2}+l^{2})}$, where the lattice spacing *d*_hkl_ = 2π/*Q*, h, k, and l are miller indices. The Gauss-Bonnet theorem establishes a relationship between the surfaced-averaged value of the Gaussian curvature (*K*) at the center of the bilayer and the lattice parameter via $K=\frac{2\pi\chi}{\sigma_{0}a^{2}}$, where *σ_0_* is the normalized surface to volume ratio and *χ* represents the interfacial topology (*σ_0_* = 1.919 and *χ* =−2 for Pn3m structure; *σ_0_* = 2.345 and *χ* = −4 for Im3m structure)[3-5]. The surface-average Gaussian curvature (*K*) can be linked to the packing properties of lipid aggregates and the transformations between particles can be quantitatively described[6]: *K* = [3/(2*l_c_*²)] * (1 - *P*), where *P* = *ν* / (*a_0_ · l_c_*) serves as a dimensionless metric for predicting lipid aggregate morphology[7], *v* represents the molecular volume of the fluid hydrocarbon chain(s), *a_0_* denotes the interfacial area per molecule at the polar–non-polar interface, and *l_c_* indicates the critical tail length. | | | |

**Supplementary Table S4. Antibacterial activity of Daptomycin/Vesicles.**

| Strains | MIC^a)^/ (µg/mL) | | FIC^b)^/ (µg/mL) | | FICI^d)^ | Outcome^e)^ |
| --- | --- | --- | --- | --- | --- | --- |
|  | Dap | LCNP^c)^ | Dap | LCNP |  |  |
| A8819 | 0.5 | > 512 | 0.25 | 64 | 0.563 | additive |
| A9719 | 0.5 | > 512 | 0.25 | 128 | 0.625 | additive |
| UK15 | 0.5 | > 512 | 0.25 | 256 | 0.750 | additive |
| A6224 | 0.5 | > 512 | 0.25 | 256 | 0.750 | additive |
| JKD6159 | 0.5 | > 512 | 0.25 | 32 | 0.531 | additive |
| A8090 | 0.5 | > 512 | 0.25 | 32 | 0.531 | additive |
| ^a)^Minimum inhibitory concentration (MIC); ^b)^Fractional inhibitory concentration (FIC); ^c)^The endpoint MIC value could not be determined due to MIC > 512 µg/mL, the next MIC value (1024 µg/mL) was used for the calculation. ^d)^FIC index (FICI) = (FICA1/MICA1) + (FICA2/MICA2), where A1 = Daptomycin (Dap) and A2 = LCNP; ^e)^Synergy was defined as FICI ≤ 0.5, additive as 0.5 <FICI < 4 and antagonism as FICI ≥4.0. All experiments were performed in triplicate (n=3). | | | | | | |

**Supplementary Table S5. Antibacterial activity of Daptomycin/P-cubosomes.**

| Strains | MIC^a)^/ (µg/mL) | | FIC^b)^/ (µg/mL) | | FICI^d)^ | Outcome^e)^ |
| --- | --- | --- | --- | --- | --- | --- |
|  | Dap | LCNP^c)^ | Dap | LCNP |  |  |
| A8819 | 0.5 | > 512 | 0.25 | 16 | 0.516 | additive |
| A9719 | 0.5 | > 512 | 0.25 | 64 | 0.563 | additive |
| UK15 | 0.5 | > 512 | 0.25 | 128 | 0.625 | additive |
| A6224 | 0.5 | > 512 | 0.25 | 128 | 0.625 | additive |
| JKD6159 | 0.5 | > 512 | 0.25 | 8 | 0.508 | additive |
| A8090 | 0.5 | > 512 | 0.25 | 8 | 0.508 | additive |
| ^a)^Minimum inhibitory concentration (MIC); ^b)^Fractional inhibitory concentration (FIC); ^c)^The endpoint MIC value could not be determined due to MIC > 512 µg/mL, the next MIC value (1024 µg/mL) was used for the calculation. ^d)^FIC index (FICI) = (FICA1/MICA1) + (FICA2/MICA2), where A1 = Daptomycin (Dap) and A2 = LCNP; ^e)^Synergy was defined as FICI ≤ 0.5, additive as 0.5 <FICI < 4 and antagonism as FICI ≥4.0. All experiments were performed in triplicate (n=3). | | | | | | |

**Supplementary Table S6. Antibacterial activity of Daptomycin/D-cubosomes.**

| Strains | MIC^a)^/ (µg/mL) | | FIC^b)^/ (µg/mL) | | FICI^d)^ | Outcome^e)^ |
| --- | --- | --- | --- | --- | --- | --- |
|  | Dap | LCNP^c)^ | Dap | LCNP |  |  |
| A8819 | 0.5 | > 512 | 0.25 | 8 | 0.508 | additive |
| A9719 | 0.5 | > 512 | 0.25 | 32 | 0.531 | additive |
| UK15 | 0.5 | > 512 | 0.25 | 64 | 0.563 | additive |
| A6224 | 0.5 | > 512 | 0.25 | 64 | 0.563 | additive |
| JKD6159 | 0.5 | > 512 | 0.25 | 4 | 0.504 | additive |
| A8090 | 0.5 | > 512 | 0.25 | 8 | 0.508 | additive |
| ^a)^Minimum inhibitory concentration (MIC); ^b)^Fractional inhibitory concentration (FIC); ^c)^The endpoint MIC value could not be determined due to MIC > 512 µg/mL, the next MIC value (1024 µg/mL) was used for the calculation. ^d)^FIC index (FICI) = (FICA1/MICA1) + (FICA2/MICA2), where A1 = Daptomycin (Dap) and A2 = LCNP; ^e)^Synergy was defined as FICI ≤ 0.5, additive as 0.5 <FICI < 4 and antagonism as FICI ≥4.0. All experiments were performed in triplicate (n=3). | | | | | | |

**Supplementary Table S7. Neutron scattering length densities (SLD).** Summary of the theoretical SLD of the substrate, lipid components, daptomycin and solution subphases used to fit neutron reflectometry data.

| Materials | SLD (× 10^-6^ Å^-2^) | | |
| --- | --- | --- | --- |
|  | D_2_O^a^ | CMSi^b^ | H_2_O^c^ |
| Si | 2.07 | 2.07 | 2.07 |
| SiO_2_ | 3.41 | 3.41 | 3.41 |
| H_2_O | - | - | -0.56 |
| CMSi | - | 2.07 | - |
| D_2_O | 6.35 | - | - |
| PG head | 3.59 | 3.09 | 2.78 |
| CL head | 3.11 | 2.99 | 2.91 |
| L-PG head | 3.78 | 2.69 | 2.02 |
| PG, CL and L-PG tails | -0.28 | -0.28 | -0.28 |
| Phytantriol | 0.37 | -0.10 | -0.39 |
| DPPS head | 3.47 | 3.47 | 3.47 |
| DPPS tail | -0.36 | -0.36 | -0.36 |
| Daptomycin | 3.96 | 3.00 | 2.41 |
| ^a^D_2_O 100%; ^b^D_2_O/H_2_O (38/62, v/v), contrast matched the silicon substrate (CMSi); ^c^H_2_O 100%. PG, 1-palmitoyl-2-oleoyl-sn-glycero-3-phospho-(1'-rac-glycerol) (sodium salt). CL, 1',3'-bis[1,2-dioleoyl-sn-glycero-3-phospho]-glycerol (sodium salt). L-PG, 1,2-dioleoyl-sn-glycero-3-[phospho-rac-(3-lysyl(1-glycerol))] (chloride salt). DPPS, 1,2-dipalmitoyl-sn-glycero-3-phospho-L-serine (sodium salt). | | | |

**Supplementary Table S8. Fitted parameters for model Gram-positive bacterial membrane bilayer treated with daptomycin (Dap)/Vesicles.**

| Sublayer | Thickness/Å | *φ*_lipid_^a^ /% | *φ*_Dap/Vesicles_^a^ /% | *φ*_hydration_^a^ /% | Roughness/Å |
| --- | --- | --- | --- | --- | --- |
| Inner head | 14.9±0.3 | 30.3±0.6 | - | 69.7±0.6 | 3.9±0.7 |
| Tail | 31.2±0.1 | 87.0±2.6 | - | 13.0±2.6 | 2.5±0.2 |
| Outer head | 8.2±0.1 | 36.7±4.7 | - | 63.3±4.7 | 3.6±0.3 |
| Daptomycin/Vesicles, 2/ 64 µg/mL | | | | | |
| Inner head | 14.9±0.1 | 27.3±2.5^b^ | | 72.7±2.5 | 3.9±0.5 |
| Tail | 31.9±0.2 | 78.7±3.5^b^ | | 21.3±3.5 | 2.7±0.4 |
| Outer head | 8.3±0.2 | 31.0±6.6^b^ | | 69.0±6.6 | 3.5±0.6 |
| ^a^Volume fraction, to represent membrane integrity. All data are expressed as median ± SD. Fitted parameters were obtained by simultaneous fitting of neutron reflectometry data across three isotopic contrasts (D₂O, CMSi, and H₂O) using the refnx analysis framework. Parameter uncertainties represent 95% confidence intervals derived from Markov Chain Monte Carlo (MCMC) analysis. As is typical for neutron reflectometry, the fitted profiles are not strictly unique. However, the use of multiple contrasts and MCMC constraints ensures physically consistent solutions and supports the reported trends. ^b^The relative volume fractions of the lipids and Dap/Vesicles in the headgroup and tail structures were not able to be determined owing to the minimal isotopic contrast (Supplementary Table S7). | | | | | |

**Supplementary Table S9. Fitted parameters for model Gram-positive bacterial membrane bilayer treated with daptomycin (Dap)/P-cubosomes.**

| Sublayer | Thickness/Å | *φ*_lipid_^a^ /% | *φ*_Dap/P-cubosomes_ ^a^ /% | *φ*_hydration_^a^ /% | Roughness/Å |
| --- | --- | --- | --- | --- | --- |
| Inner head | 14.9±0.1 | 30.3±0.6 | -  -  - | 69.7±0.6 | 3.7±0.5 |
| Tail | 31.8±0.1 | 86.0±3.6 |  | 14.0±3.6 | 3.5±0.4 |
| Outer head | 8.2±0.2 | 36.7±4.7 |  | 63.3±4.7 | 3.5±0.3 |
| Daptomycin/P-cubosomes, 2/ 64 µg/mL | | | | | |
| Inner head | 14.9±0.1 | 29.0±1.7^b^ | | 71.0±1.7 | 3.3±0.5 |
| Tail | 31.8±0.1 | 70.7±2.1^b^ | | 29.3±2.1 | 3.9±0.4 |
| Outer head | 8.2±0.2 | 28.3±2.9^b^ | | 71.7±2.9 | 3.9±0.3 |
| ^a^Volume fraction, to represent membrane integrity. All data are expressed as median ± SD. Fitted parameters were obtained by simultaneous fitting of neutron reflectometry data across three isotopic contrasts (D₂O, CMSi, and H₂O) using the refnx analysis framework. Parameter uncertainties represent 95% confidence intervals derived from Markov Chain Monte Carlo (MCMC) analysis. As is typical for neutron reflectometry, the fitted profiles are not strictly unique. However, the use of multiple contrasts and MCMC constraints ensures physically consistent solutions and supports the reported trends. ^b^The relative volume fractions of the lipids and Dap/P-cubosomes in the headgroup and tail structures were not able to be determined owing to the minimal isotopic contrast (Supplementary Table S7). | | | | | |

**Supplementary Table S10. Fitted parameters for model Gram-positive bacterial membrane bilayer treated with daptomycin (Dap)/D-cubosomes.**

| Sublayer | Thickness/Å | *φ*_lipid_^a^ /% | *φ*_Dap/D-cubosomes_ ^a^ /% | | *φ*_hydration_^a^ /% | Roughness/Å |
| --- | --- | --- | --- | --- | --- | --- |
| Inner head | 14.8±0.2 | 30.7±1.2 | -  -  - | | 69.3±1.2 | 3.5±0.9 |
| Tail | 31.3±0.2 | 84.0±3.6 |  |  | 16.0±3.6 | 3.1±0.4 |
| Outer head | 8.6±0.2 | 33.7±3.2 |  |  | 66.3±3.2 | 3.4±0.4 |
| Daptomycin/D-cubosomes, 2/ 64 µg/mL | | | | | | |
| Inner head | 14.9±0.1 | 27.0±6.1^b^ | | | 73.0±6.1 | 3.4±0.5 |
| Tail | 30.6±0.2 | 54.0±3.6^b^ | | | 46.0±3.6 | 3.0±0.3 |
| Outer head | 8.2±0.2 | 18.3±2.9^b^ | | | 81.7±2.9 | 3.4±0.3 |
| D-cubosomes | 33.5±0.9 | - | | 14.3±4.0 | 85.7±4.0 | 3.9±1.1 |
| ^a^Volume fraction, to represent membrane integrity. All data are expressed as median ± SD. Fitted parameters were obtained by simultaneous fitting of neutron reflectometry data across three isotopic contrasts (D₂O, CMSi, and H₂O) using the refnx analysis framework. Parameter uncertainties represent 95% confidence intervals derived from Markov Chain Monte Carlo (MCMC) analysis. As is typical for neutron reflectometry, the fitted profiles are not strictly unique. However, the use of multiple contrasts and MCMC constraints ensures physically consistent solutions and supports the reported trends. ^b^The relative volume fractions of the lipids and Dap/D-cubosomes in the headgroup and tail structures were not able to be determined owing to the minimal isotopic contrast (Supplementary Table S7). | | | | | | |

**References**

1. A. Nelson, "Motofit– Integrating Neutron Reflectometry Acquisition, Reduction and Analysis into One, Easy to Use, Package," *Journal of Physics: Conference Series* 251(2010): 012094, <https://doi.org/10.1088/1742-6596/251/1/012094>

2. S. M. Gruner, "Stability of lyotropic phases with curved interfaces," *The Journal of Physical Chemistry* 93, no. 22 (1989): 7562–7570, <https://doi.org/https://doi.org/10.1021/j100359a011>

3. M. Salim, W. F. N. Wan Iskandar, M. Patrick et al., "Swelling of Bicontinuous Cubic Phases in Guerbet Glycolipid: Effects of Additives," *Langmuir* 32, no. 22 (2016): 5552–5561, <https://doi.org/10.1021/acs.langmuir.6b01007>

4. R. Templer, J. Seddon, N. Warrender et al., "Inverse bicontinuous cubic phases in 2: 1 fatty acid/phosphatidylcholine mixtures. The effects of chain length, hydration, and temperature," *The Journal of Physical Chemistry B* 102, no. 37 (1998): 7251–7261, <https://doi.org/https://doi.org/10.1021/jp972835a>

5. P. Barois, D. Eidam,S. Hyde, "X-ray study of cubic phases in ternary systems of surfactant DDAB, water and oil," *Le Journal de Physique Colloques* 51, no. C7 (1990): C7–25–C27–34, <https://doi.org/https://doi.org/10.1051/jphyscol:1990703>

6. A. I. Tyler, H. M. Barriga, E. S. Parsons et al., "Electrostatic swelling of bicontinuous cubic lipid phases," *Soft Matter* 11, no. 16 (2015): 3279–3286, <https://doi.org/10.1039/c5sm00311c>

7. R. A. Khalil,A.-h. A. Zarari, "Theoretical estimation of the critical packing parameter of amphiphilic self-assembled aggregates," *Applied Surface Science* 318(2014): 85–89, <https://doi.org/10.1016/j.apsusc.2014.01.046>
